# Climate change multi-model projections in CMIP6 scenarios in Central Hokkaido, Japan

**DOI:** 10.1038/s41598-022-27357-7

**Published:** 2023-01-05

**Authors:** Shilei Peng, Chunying Wang, Zhan Li, Kunihito Mihara, Kanta Kuramochi, Yo Toma, Ryusuke Hatano

**Affiliations:** 1 Institute of Subtropical Agriculture, Chinese Academy of Sciences, Changsha, 410125 China; 2 Research Faculty of Agriculture, Hokkaido University, Sapporo, 0608589 Japan; 3 College of Water Resources, North China University of Water Resources and Electric Power, Zhengzhou, 450045 China; 4 Graduate School of Science, Hokkaido University, Sapporo, 0608589 Japan

**Keywords:** Environmental sciences, Environmental impact, Climate sciences, Atmospheric science

## Abstract

Simulation of future climate changes, especially temperature and rainfall, is critical for water resource management, disaster mitigation, and agricultural development. Based on the category-wise indicator method, two preferred Global Climate Models (GCMs) for the Ishikari River basin (IRB), the socio-economic center of Hokkaido, Japan, were examined from the newly released Coupled Model Intercomparison Project Phase 6 (CMIP6). Climatic variables (maximum/minimum temperature and precipitation) were projected by the Statistical DownScaling Model (SDSM) under all shared socioeconomic pathway-representative concentration pathway (SSP-RCP) scenarios (SSP1-1.9, SSP1-2.6, SSP2-4.5, SSP3-7.0, SSP4-3.4, SSP4-6.0, SSP5-3.4OS, and SSP5-8.5) in two phases: 2040–2069 (2040s) and 2070–2099 (2070s), with the period of 1985–2014 as the baseline. Predictors of SDSM were derived from CMIP6 GCMs and the reanalysis dataset NOAA-CIRES-DOE 20th Century Reanalysis V3 (20CRv3). Results showed that CMIP6 GCMs had a significant correlation with temperature measurements, but could not represent precipitation features in the IRB. The constructed SDSM could capture the characteristics of temperature and precipitation during the calibration (1985–1999) and validation (2000–2014) phases, respectively. The selected GCMs (MIROC6 and MRI-ESM-2.0) generated higher temperature and less rainfall in the forthcoming phases. The SSP-RCP scenarios had an apparent influence on temperature and precipitation. High-emission scenarios (i.e., SSP5-8.5) would project a higher temperature and lower rainfall than the low-emission scenarios (e.g., SSP1-1.9). Spatial–temporal analysis indicated that the northern part of the IRB is more likely to become warmer with heavier precipitation than the southern part in the future. Higher temperature and lower rainfall were projected throughout the late twenty-first century (2070s) than the mid-century (2040s) in the IRB. The findings of this study could be further used to predict the hydrological cycle and assess the ecosystem's sustainability.

## Introduction

The current rate and scale of global warming are exceptional with respect to the pre-industrial age^1^. It is increasingly evident that climate change will drive longer and more vigorous-intensity to extremes with severe impacts on humanity^2^, economy^3^, and natural ecosystems^4,5^. Limiting global temperature increases to 1.5 °C is vital to stave off the worst warming climate-related risks^6,7^. As a response, it is critical to investigate possible variations in future climatic variables (such as air temperature and precipitation), which should be a major issue for stakeholders to manage regional catastrophic hazards, prevent significant consequences, as well as establish adaptation plans. Due to the constraints in studies of historical fluctuations and known trends, climate forecasts are required for decision-support modeling^8^. The World Climate Research Program (WCRP) Coupled Model Intercomparison Project (CMIP) provides one of the most advanced tools, the Global Climate Models (GCMs). GCMs are commonly employed in studies to reproduce physical processes in the atmosphere, ocean, terrestre, and cryosphere. They also provide feedback on global or continental climatic changes under various emission scenarios^9^. The Fourth and Fifth Assessment Reports of the Intergovernmental Panel on Climate Change, IPCC AR4 and AR5, exhibit and evaluate different generations of GCM outputs of CMIP3 and CMIP5^10,11^. However, lots of studies pointed out there are limitations in previous CMIP3- or CMIP5-based GCMs^12,13^. Those products lack of complete information about atmospheric-climatic processes, leading to significant uncertainties and climate sensitivities^14–16^. The latest CMIP6 phase aims to improve the mechanism of emission scenarios and increase the horizontal resolution, making future possibilities more plausible^17,18^.

Nevertheless, the performance of GCMs for simulating regional climatic variables still does not satisfy the accuracy requirement of practitioners^19^. Hence, it needs to fabricate local-scale daily climatic conditions by applying a downscaling approach. Statistical downscaling approaches have become the main strategy for inferring regional information from coarse GCMs. They play the role of “bridge” to connect the large scale and local areas^20,21^. Statistical downscaling strategies also have the irreplaceable aspects to provide information that other methodological approaches (e.g., dynamical downscaling) cannot give, even as developing new generation GCMs with higher resolution^20^. The Statistical Downscaling Model (SDSM) is the preferred approach to eliminate errors from GCMs^22^. Lots of SDSM-related studies have demonstrated SDSM's ability to generate future change scenarios^23^. However, only two common GCMs (CanESM2 and HadCM3) were widely employed to access climatic variables in SDSM. For example, Gebrechorkos et al. utilized SDSM to draw future temperature and rainfall changes (only CanESM2) in East Africa (Ethiopia, Kenya, and Tanzania)^24^. Emami and Koch (2019) employed SDSM to reveal the influence of temperature change (only CanESM2) on water resources in a mountainous area from Iran^25^. Phuong et al. utilized SDSM to reproduce future climatic variables (CanESM2 and HadCM3) on the daily scale under RCP scenarios in a river basin of mid-Vietnam^26^.

Meanwhile, several downscaling-related studies have largely ignored the assessment of GCMs adaptability to reduce the uncertainty and GCM selection in specific study locations. A rigorous evaluation of GCMs before they are applied in hydrology or agricultural management might boost stakeholders’ confidence in using GCMs^27^. Wilby and Harris created a probabilistic approach to overcome CMIP3 GCM conflicts about regional climatic changes in the River Thames, the United Kingdom^28^. Gleckler et al. created a set of measurements to accurately measure the relative advantages of CMIP3 models^29^. Aloysius et al. assessed 25 CMIP5 GCMs in Central Africa regarding historical performance, intermodel and future emission scenario uncertainties^30^. Wang et al. scored 23 CMIP5 GCMs in an inland basin of Northwest China for the future projections of temperature and precipitation^31^. With the continuous improvement of released CMIP6, several researchers have studied subsets of CMIP6 GCMs, resulting in varied model downscaling. For example, Kreienkamp et al. downscaled CMIP6 GCM outputs in Germany using the statistical-empirical downscaling approach^32^. Chaudhuri and Robertson developed the deep neural network model with a structural sensitivity to downscale large-scale annual maximum precipitation from 9 CMIP6 GCMs in Great Bear Lake in Northwest Territories, Canada^33^. However, few studies on SDSM of temperature and precipitation based on CMIP6 outputs have been undertaken.

Hence, in this study, all available CMIP6-GCMs (a total of 17 until November 2021) were assessed by comparing them to observed climatic data, and then downscaled by SDSM across the Ishikari River basin (IRB), which is the most socioeconomically significant basin in Hokkaido, Japan. The primary goals of this study were (1) to select preferred CMIP6 GCMs for the IRB to reduce the uncertainties; (2) to re-establish SDSM predictors between reanalysis datasets of NOAA-CIRES-DOE 20th Century Reanalysis V3 (20CRv3) and CMIP6 GCMs; (3) to project future changes in climatic variables (temperature and precipitation) across the IRB during the mid twenty-first century (2040–2069, 2040s) and late twenty-first century (2070–2099, 2070s) under all SSP-RCP scenarios from the CMIP6 GCMs, compared with the baseline period (1985–2014).

## Materials and methods

### Study area

**Figure 1:**
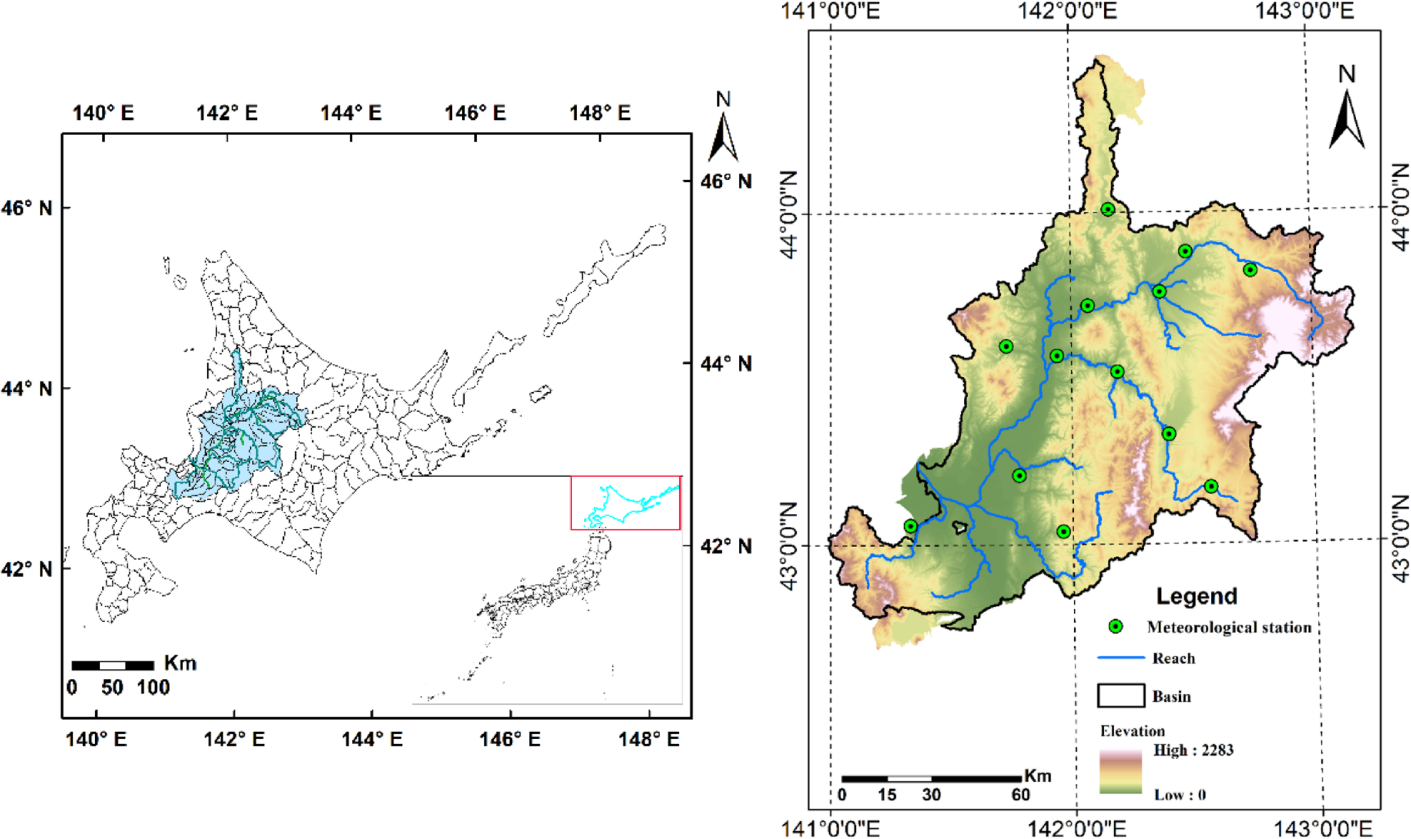
Locations of the Ishikari River basin (IRB) and its meteorological stations. Note: This figure was generated by ArcMap 10.2 (https://support.esri.com/en/products/desktop/arcgis-desktop/arcmap/10-2-2). Shapefiles are available from the Japanese Geographical Survey Institute (JGSI, http://nlftp.mlit.go.jp).

The Ishikari River basin (IRB, 42°41′9.6″N–44°47′8.9″N, 140°59′33″E–143°10′47.8″E) was situated in the Mid-western Hokkaido, Japan, with an area of 14,330 km^2^ (Fig. 1). The Ishikari River originates from Mt. Ishikari and flows westward to the Sea of Japan. Toyohira, Tobetsu, Chitose, and Yubari are its major tributaries^34^. 52% of the population of Hokkaido live in the IRB. The IRB is an important economic, agricultural, industrial, and cultural center of Hokkaido, and is also the seat of Sapporo and Asahikawa, the largest two cities of Hokkaido. The IRB is dominantly controlled by the hot-summer subtype and hemiboreal climate. According to the climate records (1985–2014) of 13 meteorological stations across the IRB provided by the Japan Meteorological Agency (JMA, http://www.jma.go.jp), the annual average maximum and minimum temperatures are 10.27–12.74 °C and 0.34–5.67 °C, respectively. The annual precipitation is about 1007–1610 mm. The rainy season is generally from August to September. The snowfall period is from mid-December to late March of the following year, with an average annual maximum snow depth of 35 cm. Hydrologic peaks occur during the snow-melt period (March to May).

### Data collection

Three types of meteorological datasets were employed in this study, including observed historical data, reanalysis data, and GCM data. IRB covered 13 meteorological stations (as shown in Fig. 1). The historical meteorological data across 30 years period of 1985–2014 was composed of daily maximum air temperature at 2 m (tasmax), daily minimum air temperature at 2 m (tasmin), and daily precipitation (pr) of each meteorological station, which can be accessed in the JMA.

In this study, the reanalysis dataset selected the most recent version of reanalysis from the Twentieth Century Reanalysis (20CR) Project, which is funded by the National Oceanic and Atmospheric Administration (NOAA), the Cooperative Institute for Research in Environmental Sciences (CIRES), and the U.S. Department of Energy (DOE), NOAA-CIRES-DOE 20th Century Reanalysis V3 (20CRv3). The 20CRv3 contained objectively-analyzed 4-dimensional weather maps and their uncertainties^35^. The 20CRv3 covered the spatial resolution of 1.0-degree latitude × 1.0-degree longitude global grid (360 × 181). Daily atmospheric variables (such as humidity, precipitation flux, and geopotential height) of 20CRv3, spanning 1985 to 2014, were readily accessible at the NOAA Physical Sciences Laboratory (PSL, https://www.psl.noaa.gov/).

Monthly and daily GCM datasets under the first variant level “r1i1p1f1” were obtained from the Coupled Model Intercomparison Project Phase 6 (CMIP6, https://esgf-node.llnl.gov/search/cmip6/). According to the availability of predictors, 17 GCMs were selected in this study, as shown in Table 1. All these predictors were interpolated onto a 1° × 1° grid to match the 20CRv3. The GCMs-derived monthly variables (tasmax, tasmin and pr) in the period of 1985–2014 were applied for the selection of GCMs.

**Table 1:** Available CMIP6 GCMs sources and information.

No	GCMs	Institution	Nominal resolution (km)
1	AWI-CM-1-1-MR	Alfred Wegener Institute, Helmholtz Centre for Polar and Marine Research, Germany	100
2	AWI-ESM-1-1-LR		250
3	FGOALS-g3	Chinese Academy of Sciences, China	250
4	CanESM5	Canadian Centre for Climate Modelling and Analysis, Environment and Climate Change Canada, Canada	500
5	CMCC-ESM2	Fondazione Centro Euro-Mediterraneo sui Cambiamenti Climatici, Italy	100
6	ACCESS-ESM1-5	Commonwealth Scientific and Industrial Research Organisation, Australia	250
7	ACCESS-CM2	Commonwealth Scientific and Industrial Research Organisation, Australia & ARCCSS (Australian Research Council Centre of Excellence for Climate System Science)	250
8	FIO-ESM-2-0	FIO (First Institute of Oceanography, Ministry of Natural Resources, China) & QNLM (Qingdao National Laboratory for Marine Science and Technology, China)	100
9	MIROC6	JAMSTEC (Japan Agency for Marine-Earth Science and Technology, Japan) & AORI (Atmosphere and Ocean Research Institute, The University of Tokyo, Japan) & NIES (National Institute for Environmental Studies, Japan) & R-CCS (RIKEN Center for Computational Science, Japan)	250
10	MPI-ESM-1-2-HAM	Max Planck Institute for Meteorology, Germany	250
11	MPI-ESM1-2-HR		100
12	MPI-ESM1-2-LR		250
13	MRI-ESM2-0	Meteorological Research Institute, Japan	100
14	GISS-E2-1-G	Goddard Institute for Space Studies, USA	250
15	GISS-E2-1-H		250
16	GISS-E2-2-H		250
17	NESM3	Nanjing University of Information Science and Technology, China	250

The daily atmospheric predictors (corresponding to 20CRv3), spanning 1985–2100, were applied in statistical downscaling analysis to generate and project future climatic variables under eight Shared Socioeconomic Pathways-Representative Concentration Pathways (SSP-RCPs) scenarios (SSP1-1.9, SSP1-2.6, SSP4-3.4, SSP2-4.5, SSP4-6.0, SSP3-7.0, SSP5-3.4OS, and SSP5-8.5). CMIP6 used a matrix framework that combined two determinants of emission scenarios (e.g., RCPs) and a diverse range of socioeconomic assumptions, namely the so-called Shared Socioeconomic Pathways (SSPs) scenarios, to force climate models^17,36,37^, which makes future scenarios more reasonable. The growth of civilization and natural systems at the national and regional levels in the twenty-first century provides a foundation for the formation of SSPs^38^. Five narratives of SSP (SSP1, SSP2, SSP3, SSP4, and SSP5) scenarios were employed to picture the potential challenges brought about by the variations in global and regional evolution across time^39,40^. Numbers are consistent with low-to-high concerns in mitigation and adaptation in the futuristic society, named SSP1 (“taking the green road”), SSP2 (“a middle of the road”), SSP3 (“regional rivalry—a rocky road”), SSP4 (“inequality—a road divided”), and SSP5 (“fossil-fueled development”), respectively^41–43^.

### Evaluation of GCMs performance

GCMs are extensively applied to simulate past climates and produce future climatic variables^44^. However, there is a significant uncertainty in estimating regional applications of GCMs due to the difference in each GCM, such as resolution (fine or coarse), climatic response mechanism (aerosols, circulations of land, ocean, and atmosphere), and spatial–temporal scales^45^. Hence, there is an urgent need to analyze each chosen GCM to minimize the uncertainties when applying them in specific areas. Evaluating the performance of GCMs simulation is generally to compare them with reanalysis or observed climatic data. Lots of indicators have been employed by various researchers in climate modelling. Raju and Kumar reviewed more than hundreds of works on climate models to study which are the best GCMs^46^. They recommended using category-wise indicators when evaluating GCMs, such as error and correlation coefficient. The Taylor diagram can compare simulations (model) with measurements using the correlation coefficient, root-mean-square difference, and standard deviations to graphically assess these qualities^47^. The high correlation and few errors represent that selected GCMs are suitable for the local climate system. Therefore, the Taylor diagram was used in this work to compare CMIP6 outputs with regional data and further to identify the best CMIP6 GCMs for modeling temperature and precipitation across the IRB.

### The statistical downscaling model

#### Description

SDSM, designed by Wilby et al. is a decision-making support tool for analyzing the implications of local climate changes^22^. SDSM 4.2, based on the Visual Basic programme, was widely used in many climate-related investigations^23^. SDSM can set up statistical relationships between large-scale predictors and regional-scale climatic conditions (e.g., temperature and precipitation) using a combination of multiple linear regressions. If these correlations remain true as in prospective, they may be utilized to acquire downconverted regional features in certain coming phases by forcing the interactions with GCM-derived predictors through the stochastic weather generator. There are two different progressions in each sub-model, unconditional and conditional. Temperature does not need to be transformed and directly generated in the unconditional pattern, which exhibits a linear relationship between the predictors and predictand (e.g., individual wind speeds can be used to calculate regional airflow parameters). Precipitation should be reformed by the fourth root and then simulated in the conditional pattern, which is an intermediate process between regional forcing and local climatic conditions. For instance, local precipitation is determined by the occurrence of rainy days, while the latter is determined by regional-scale predictors (such as moisture and atmospheric pressure). The wet criterion for daily rainfall was chosen at 1.0 mm in this study, as it is commonly employed for statistical downscaling.

#### Model process

SDSM performs five key steps, from variables selection, calibrating and validating model, to weather generation and future climate scenarios projection^48^. Screening variables between predictand (such as maximum temperature, minimum temperature, evaporation, as well as precipitation on a local scale) and predictor (large-scale atmospheric conditions) is a core of the statistical downscaling process. SDSM combines the correlation matrix, partial correlation, P value, histograms, and scatter plots, which can help users to find the best predictors. Peng et al. provided extensive explanations of each SDSM procedure^49^.

In this study, predictors were constructed by the reanalysis datasets (20CRv3) and CMIP6 GCMs to reproduce ensembles of present climate data in SDSM. Commonly used predictors are normalized and obtained as predictor datasets. In this study, predictors are comprised of mean temperature at 2 m (temp), mean sea level pressure (mslp), total precipitation (prcp), surface downwelling longwave flux in air (rlds) and surface downwelling shortwave flux in air (rsds) in monolevel, specific humidity (#_shum), relative humidity (#_rhum), geopotential height (_p), geostrophic air flow velocity (#_f), vorticity (#_z), zonal velocity component (#_u), meridional velocity component (#_v), divergence (#_zh), and wind direction (#_th) under the pressure level. Table 2 lists the most suitable predictors for observed predictands in 13 meteorological stations across the IRB. Simulation of precipitation needs more predictors other than temperature.

**Table 2:** List of selected predictors in the Ishikari River basin.

Stations	Predictand		
	Maximum air temperature (tasmax)	Minimum air temperature (tasmin)	Precipitation (pr)
Horokanai	1000 shum, temp, 500 p	1000 shum, temp, rlds	1000 u, rsds, 700 p, 850 rhum
Pippu	1000 shum, temp, 500 p	1000 shum, temp, rlds	1000 u, rsds, 700 p, 800 p, 850 rhum, 1000 rhum
Kamikawa	1000 shum, temp, 500 p	1000 shum, temp	rsds, 700 p, 800 p, 850 rhum
Asahikawa	1000 shum, temp, 500 p	1000 shum, temp, rlds	1000 u, rsds, 700 p, 850 rhum
Fukagawa	1000 shum, temp, 500 p	1000 shum, temp, rlds	1000 u, rsds, 700 p,850 rhum,
Sorachiyoshino	1000 shum, temp, 500p	1000shum, temp, rlds	1000 u, rsds, 700 p, 800 p, 850 rhum
Takikawa	1000shum, temp, 500 p	1000 shum, temp	1000 u, rsds, 700 p, 850 rhum
Ashibetsu	1000 shum, temp, 500 p	1000 shum, temp	rsds, 700 p, 850 rhum
Furano	1000 shum, temp, 500 p	1000 shum, temp	rsds, 700 p, 850 p, 850 rhum
Iwamizawa	1000 shum, temp, 500 p	1000 shum, temp	1000 u,700 p, 850 p, 850 rhum, prcp
Ikutora	1000 shum, temp, 500 p	1000 shum, temp	1000 u, rsds, 700 p, 850 p, 850 rhum, prcp
Sapporo	1000 shum, temp, 500 p	1000 shum, temp	700 p, 850 p, 7000 rhum, 850 rhum, prcp
Yubari	1000 shum, temp, 500 p	1000 shum, temp	rsds, 700 p, 850 p, 850 rhum

Mean temperature at 2 m (temp), mean sea level pressure (mslp), total precipitation (prcp), surface downwelling longwave flux in air (rlds) and surface downwelling shortwave flux in air (rsds) in monolevel, specific humidity (#_shum), relative humidity (#_rhum), geopotential height (_p), geostrophic air flow velocity (#_f), vorticity (#_z), zonal velocity component (#_u), meridional velocity component (#_v), divergence (#_zh), and wind direction (#_th) under the pressure level of 500 hPa, 700 hPa, 850 hPa, as well as 1000 hPa.

#### SDSM evaluation

Generally speaking, the model’s performance is mainly based on the selection step for predictors to predictands in SDSM. Even though there are statistical or graphical ways, such as correlation matrix and P value during the screening process, to identify the most accurate predictors, applying statistical parameters in the evaluation process is necessary to avoid uncertainties in SDSM. The ability of modeling outputs from SDSM was obtained by determining the Nash and Sutcliffe efficiency [NSE, Eq. (1)], coefficient of determination [R^2^, Eq. (2)], root mean square error [RMSE, Eq. (3)], and percent bias [P_bias,_ Eq. (4)].

The magnitude of NSE is computed using the formula below:

1 $$NSE=1-\frac{\sum_{i=1}^{n}{\left({X}_{oi}-{X}_{mi}\right)}^{2}}{\sum_{i=1}^{n}{\left({X}_{oi}-{\overline{X}}_{mi}\right)}^{2}}$$

The magnitude of R^2^ is calculated using the following equation:

2 $${\mathrm{R}}^{2}=\frac{\sum_{i=1}^{n}\left({X}_{oi}-{\overline{X}}_{oi}\right)\left({X}_{mi}-{\overline{X}}_{mi}\right)}{[\sum_{i=1}^{n}({{{X}_{oi}-{\overline{X}}_{oi})}^{2}]}^{0.5}[\sum_{i=1}^{n}({{{X}_{mi}-{\overline{X}}_{mi})}^{2}]}^{0.5}}$$

The magnitude of RMSE is computed using the formula below:

3 $$RMSE=\sqrt{\frac{\sum_{i=1}^{N}{\left({X}_{oi}-{X}_{mi}\right)}^{2}}{N}}$$

The magnitude of Pbias is calculated using the following equation:

4 $${P}_{bias}=\frac{\sum_{i=1}^{N}({X}_{mi}-{X}_{oi})}{\sqrt{\sum_{i=1}^{N}{X}_{oi}}}\times 100$$

where ${X}_{oi}$ is the observed predictand on day i, ${X}_{mi}$ is the modeling outcome on day i, $\overline{X }$_oi_ is the average measured value during the study period, and n is the total number of the observed data. The NSE illustrates how well the observed and simulated data suit the 1:1 line. Both R^2^ and *RMSE* are indices of quality of fit, whereas *Pbias* reveals the model’s tendency to over- or under-estimated with respect to the observed data. The model performs well when R^2^ and NSE values are close to one, and the lower the RMSE and absolute value of Pbias are, the tighter the modeled and measured magnitudes are^50^.

## Results

### Selection of GCMs

**Figure 2:**
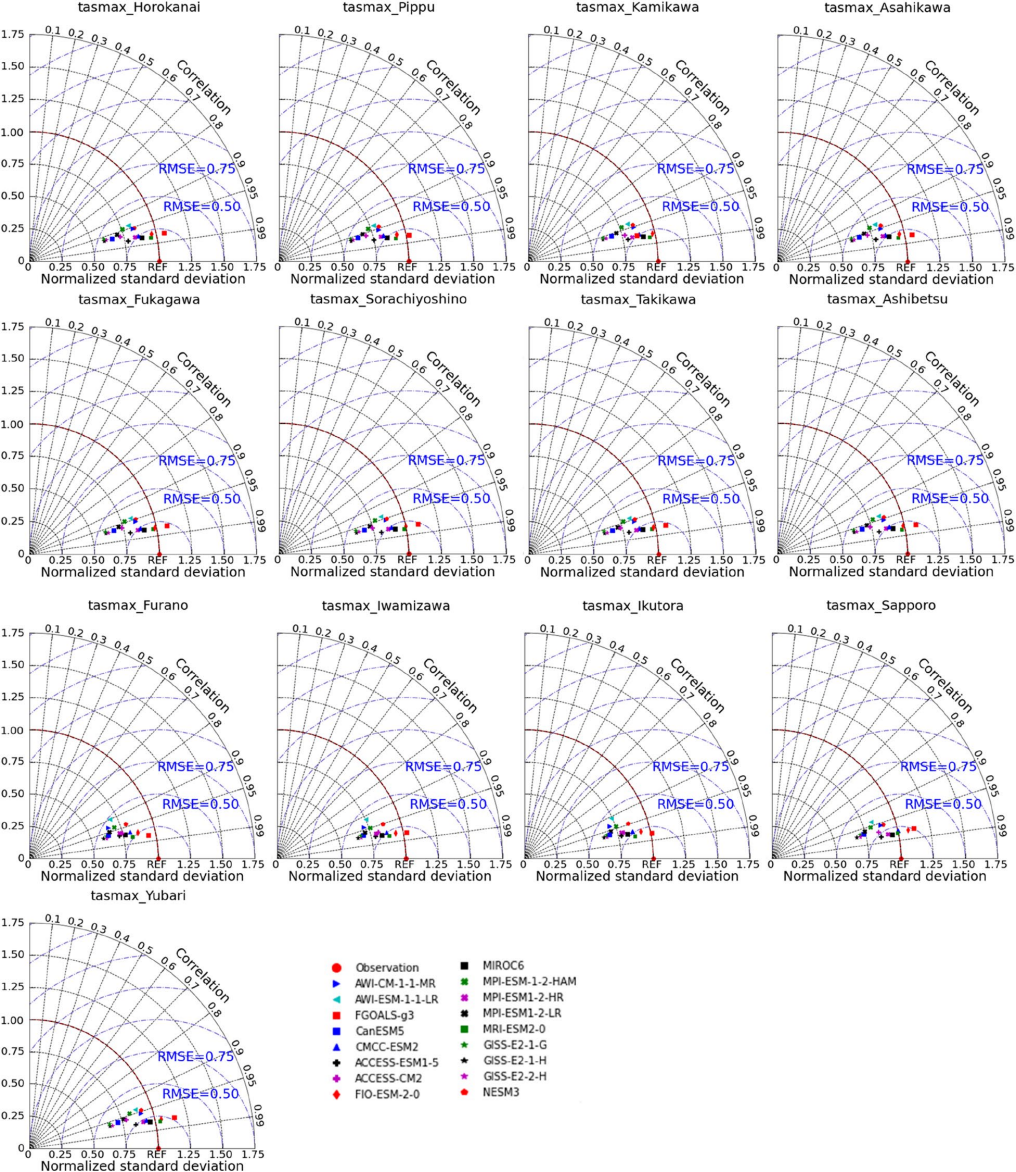
Taylor diagrams of maximum air temperature (tasmax) for each GCM at each station.

**Figure 3:**
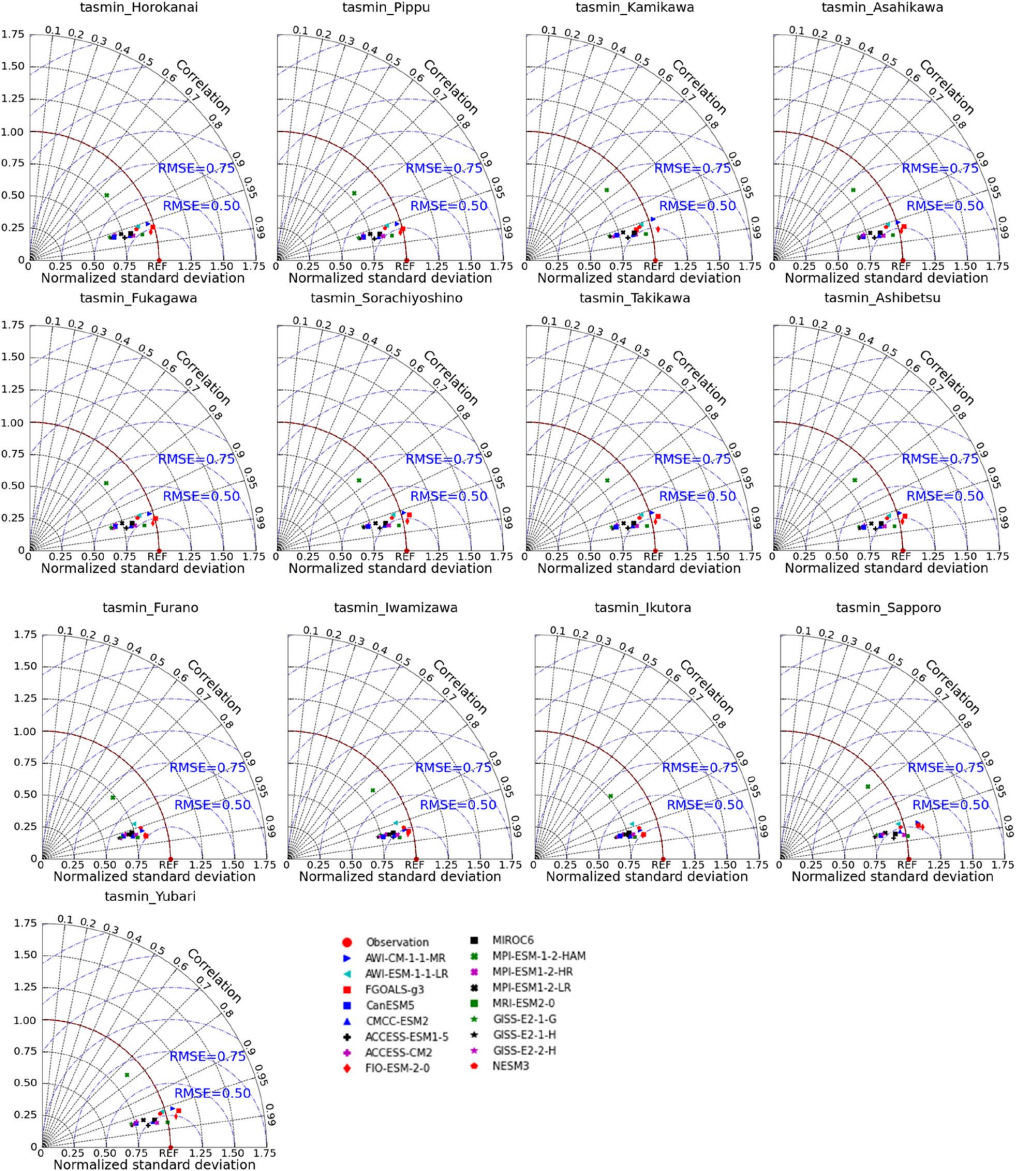
Taylor diagrams of minimum air temperature (tasmin) for each GCM at each station.

**Figure 4:**
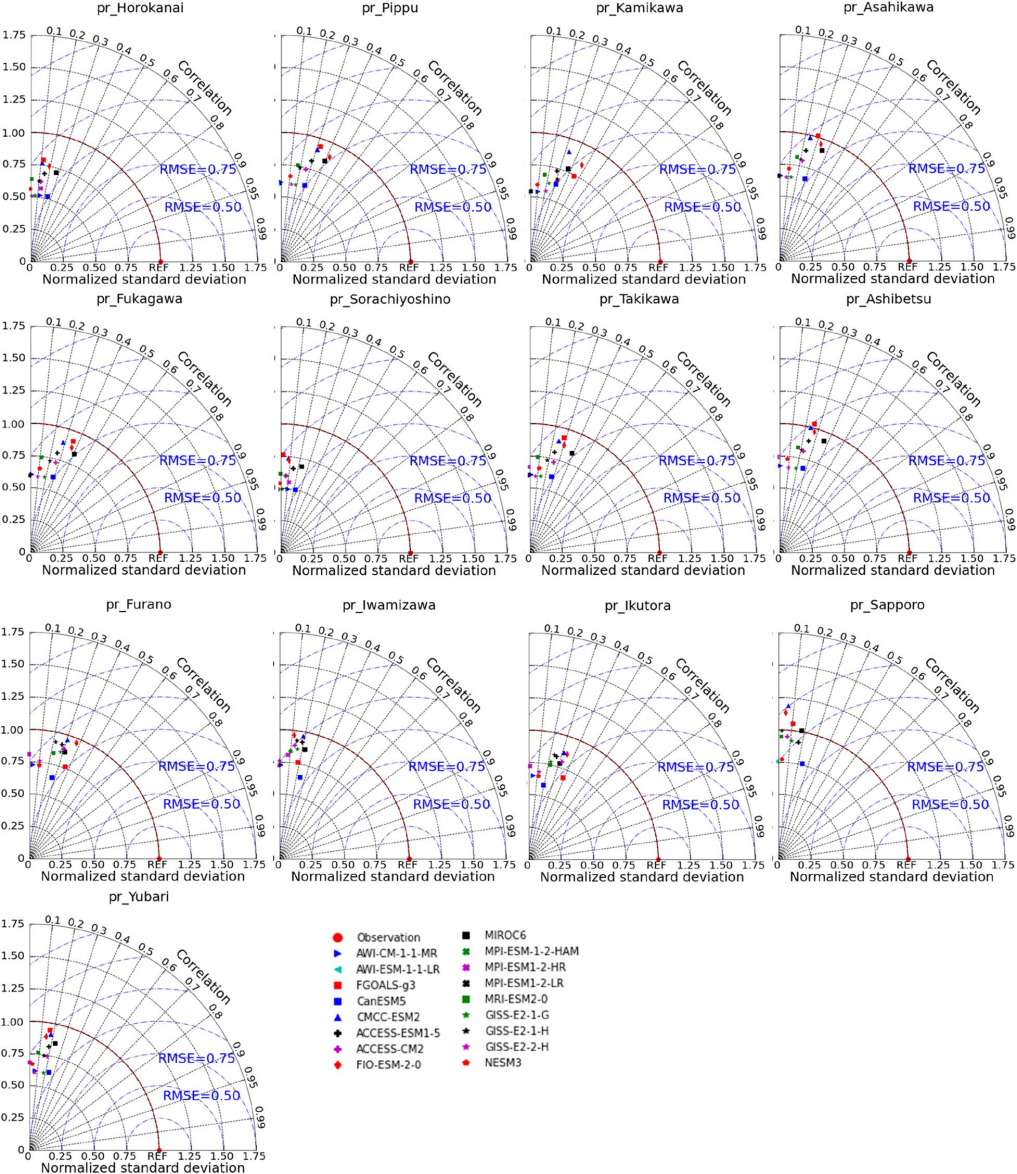
Taylor diagrams of precipitation (pr) for each GCM at each station.

When assessing GCMs, it is vital to compare GCM outputs with observed records; otherwise, even previous robust predictions may not provide skilled future projections^51^. The observed historical climatic variables were compared to modelled datasets derived from 17 GCMs on the 'historical' experiments throughout 1985–2014, as shown in Figs. 2, 3, and 4, which are presented by Taylor diagrams of maximum air temperature (tasmax), minimum air temperature (tasmin), as well as precipitation (pr) on the monthly scale at 13 stations. The relative position of GCM points (distance from red dot) on the Taylor diagrams could be used to select appropriate GCMs for each meteorological station. GCMs with high correlations and few errors showed better simulation effects when compared with observed values. For example, at Horokanai, Kamikawa, Furano, and Sapporo stations, in terms of tasmax (Fig. 2), GCMs such as FGOALS-g3, ACCESS-ESM1-5, MIROC6, and MRI-ESM2-0 (having a correlation coefficient of 0.979, 0.979, 0.978, and 0.982, respectively) were close to observations in Horokanai. MIROC6 and MRI-ESM2-0 performed the highest correlation values in Kamikawa (MIROC6: 0.978, MRI-ESM2-0: 0.981) and Furano (MIROC6: 0.981, MRI-ESM2-0: 0.978). The correlation of FGOALS-g3, ACCESS-ESM1-5, MIROC6 and MRI-ESM2-0 in Sapporo was 0.978, 0.980, 0.980, and 0.980, respectively, revealing a strong connection between GCMs and observed station data. GCMs such as FIO-ESM-2-0 and MRI-ESM2-0 had the value of RMSE lower than 0.25 when assessing *tasmin* in Horokanai and Kamikawa (Fig. 3). FGOALS-g3 and MRI-ESM2-0 had the best correlation with tasmin in Furano with a magnitude of 0.976 and 0.975, respectively. The highest correlation magnitudes happened on such GCMs as ACCESS-ESM1-5 (0.983) and MRI-ESM2-0 (0.983) when comparing the tasmin in Sapporo. However, all the GCMs could not capture the precipitation well (Fig. 4). Among 17 GCMs, MIROC6 exhibited the best modeling effects, which was closest to the observations, as shown in Fig. 4. The correlations of MIROC6 in Horokanai, Kamikawa, Furano, and Sapporo were low at 0.270, 0.370, 0.312, and 0.176, respectively. For each of the climatic variables from 17 CMIP6 GCMs in the study area, similar outputs were obtained from other stations (Figs. 2, 3, and 4).

### SDSM downscaling

In this study, the span of 1985–1999 was considered as the calibrated phase, and the validated phase was from 2000 to 2014. Table 3 lists the values of four evaluation metrics (such as R^2^, RMSE, NSE, and Pbais) at 13 meteorological stations when calibrating and validating maximum/minimum air temperature and precipitation on a monthly scale. The R^2^ and NSE values of each meteorological station's maximum or minimum air temperatures throughout the simulation phase were nearly equal to 1.000. In terms of maximum air temperature, the values of RMSE in these two modeling phases ranged within 0.035–1.065 and 0.089–0.964, respectively. The Pbias values were less than 0.400% in the calibration phase, with the exception at the Horokanai station (4.653%), and were less than 1.000% in the validation phase, with the exception at the Horokanai station (1.374%). When simulating the minimum air temperature, RMSE values in 1985–1999 and 2000–2014 were 0.034–0.123 and 0.112–0.274, respectively. The absolute values of Pbias were 0.03–9.84% in the downscaling phases. Satisfactory metric ranges revealed that SDSM could well simulate maximum/minimum temperatures across the targeted basin.

**Table 3:** SDSM-based evaluation metrics of each station during the phases of calibration (1985–1999) and validation (2000–2014).

Periods	Predictand	Evaluation index	Meteorological station												
			Horokanai	Pippu	Kamikawa	Asahikawa	Fukagawa	Sorachiyoshino	Takikawa	Ashibetsu	Furano	Iwamizawa	Ikutora	Sapporo	Yubari
Calibration (1985–1999)	Maximum temperature	R^2^	0.997	1.000	1.000	1.000	1.000	1.000	1.000	1.000	1.000	1.000	1.000	1.000	1.000
		RMSE	1.065	0.051	0.035	0.044	0.057	0.038	0.045	0.046	0.078	0.081	0.073	0.062	0.036
		Pbias (%)	4.653	0.092	− 0.012	− 0.056	− 0.031	0.084	− 0.011	− 0.025	0.239	− 0.217	− 0.352	− 0.058	0.054
		NSE	0.988	1.000	1.000	1.000	1.000	1.000	1.000	1.000	1.000	1.000	1.000	1.000	1.000
	Minimum temperature	R^2^	1.000	1.000	1.000	1.000	1.000	1.000	1.000	1.000	1.000	1.000	1.000	1.000	1.000
		RMSE	0.088	0.043	0.052	0.075	0.035	0.040	0.058	0.052	0.088	0.123	0.099	0.035	0.034
		Pbias (%)	0.490	0.612	− 1.773	0.341	0.312	− 0.896	0.284	0.214	− 0.948	1.661	9.837	− 0.044	− 0.036
		NSE	1.000	1.000	1.000	1.000	1.000	1.000	1.000	1.000	1.000	1.000	1.000	1.000	1.000
	Precipitation	R^2^	0.991	0.988	0.980	0.987	0.981	0.991	0.994	0.995	0.994	0.987	0.996	0.992	0.989
		RMSE	6.126	5.450	6.444	4.561	5.979	6.849	4.962	4.125	5.212	5.988	5.567	5.710	6.461
		Pbias (%)	− 3.259	− 3.935	− 3.310	− 2.994	− 3.992	− 3.712	− 3.810	− 3.651	− 4.063	− 4.531	− 4.582	− 5.025	− 4.331
		NSE	0.997	0.995	0.995	0.997	0.994	0.997	0.997	0.998	0.995	0.995	0.995	0.995	0.996
Validation (2000–2014)	Maximum temperature	R^2^	0.998	1.000	1.000	1.000	1.000	1.000	1.000	1.000	1.000	1.000	1.000	1.000	1.000
		RMSE	0.964	0.125	0.097	0.119	0.102	0.098	0.089	0.103	0.142	0.119	0.140	0.111	0.120
		Pbias (%)	1.374	0.197	0.242	0.328	0.151	0.010	0.104	0.165	0.805	0.088	0.988	0.278	0.190
		NSE	0.991	1.000	1.000	1.000	1.000	1.000	1.000	1.000	1.000	1.000	1.000	1.000	1.000
	Minimum temperature	R^2^	1.000	1.000	1.000	1.000	0.999	1.000	1.000	1.000	0.999	1.000	1.000	1.000	1.000
		RMSE	0.220	0.209	0.199	0.197	0.238	0.192	0.178	0.205	0.274	0.154	0.222	0.112	0.149
		Pbias (%)	0.923	1.752	0.926	0.432	− 0.570	− 0.282	0.229	− 0.030	2.582	− 0.512	− 0.130	− 0.031	0.497
		NSE	1.000	1.000	1.000	1.000	1.000	1.000	1.000	1.000	1.000	1.000	1.000	1.000	1.000
	Precipitation	R^2^	0.962	0.986	0.984	0.970	0.971	0.974	0.957	0.977	0.980	0.974	0.961	0.983	0.972
		RMSE	9.175	5.751	7.318	6.967	7.916	9.489	8.765	6.215	6.659	6.580	9.986	12.768	19.497
		Pbias (%)	− 1.794	− 3.366	− 3.081	− 3.087	− 2.595	− 3.707	− 1.887	− 2.297	− 3.839	− 3.431	− 4.331	11.441	14.336
		NSE	0.994	0.996	0.994	0.993	0.992	0.995	0.991	0.995	0.993	0.995	0.988	0.984	0.978

As accessing the precipitation, the R^2^ magnitudes (all 13 stations) ranged from 0.980 to 0.996 during the calibration phase and from 0.957 to 0.986 during the validation phase. The values of RMSE spanned from 4.13 to 19.50 in these two modeling phases. The NSE values in both two phases were greater than 0.978. The absolute values of Pbias in these two phases were 2.99–5.02% and 1.79–14.34%, respectively. Only the Pbias of precipitation at the Yubari station reached 14.34% during the validation phase. The downscaling progress in SDSM during the calibration phase performed better than that in the validation phase. Meanwhile, the outcomes demonstrated that SDSM outperforms precipitation in modeling tasmax and tasmin. Precipitation is always difficult to simulate due to its high dynamic properties^52^. Generally, choosing an effective mix of predictors for SDSM is challenging owing to influencing variables such as dry and wet period durations, local microclimates, and terrains, which could not be fully covered in reanalysis datasets^53^. In this study, results in downscaling temperature and precipitation demonstrated that predictors from the 20CRv3 dataset are able to reflect attributes on a local scale. In total, SDSM performs effectively in simulating the climatic variables during both calibration and validation phases in the IRB.

### Analysis of climatic variables at each station

**Figure 5:**
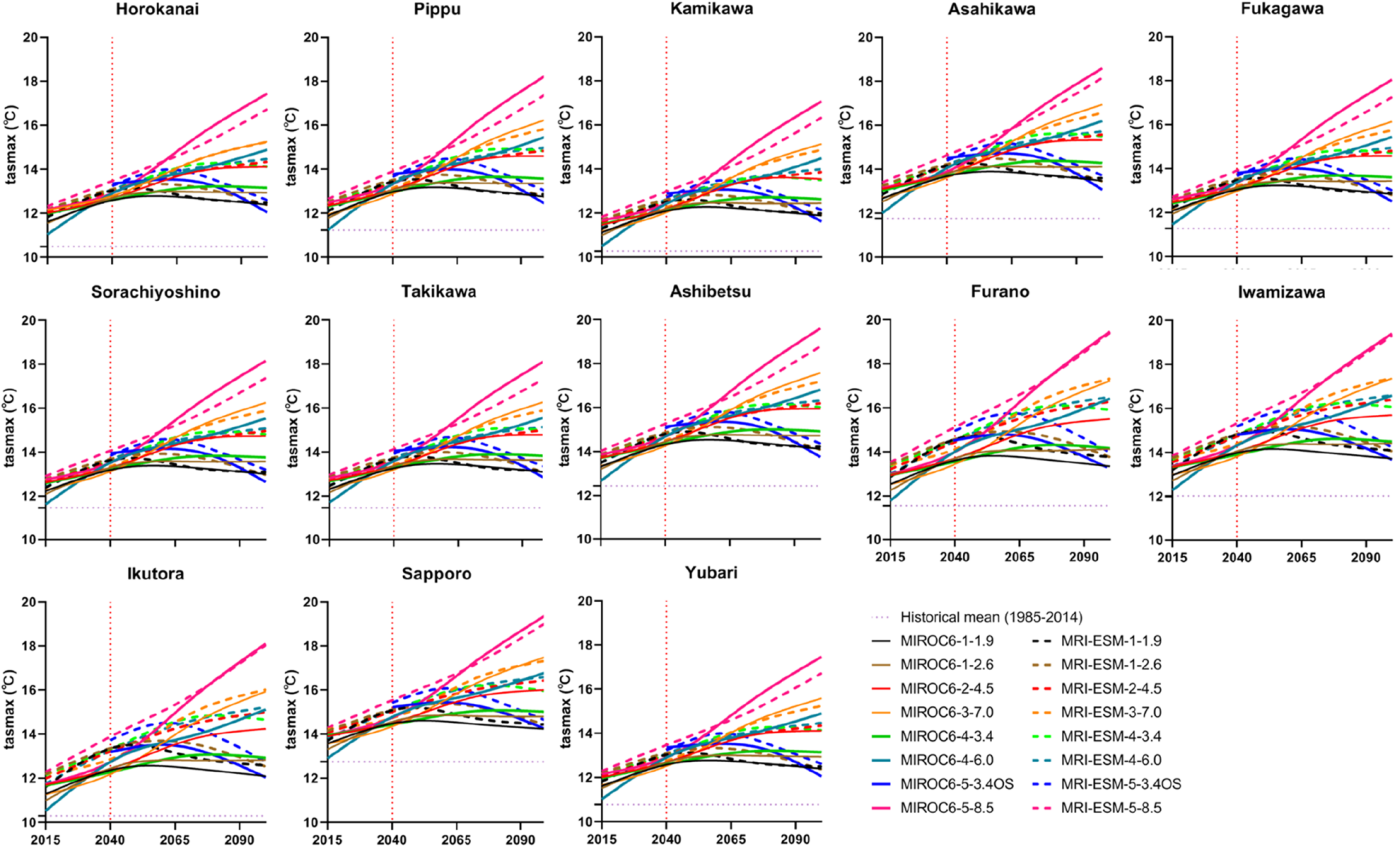
Annual mean maximum temperature (tasmax) under SSP-RCPs scenarios of two GCMs.

**Figure 6:**
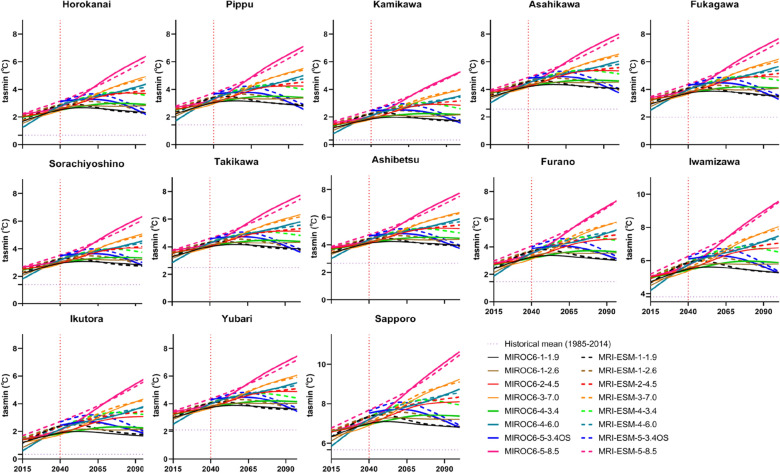
Annual mean minimum temperature (tasmin) under SSP-RCPs scenarios of two GCMs.

**Figure 7:**
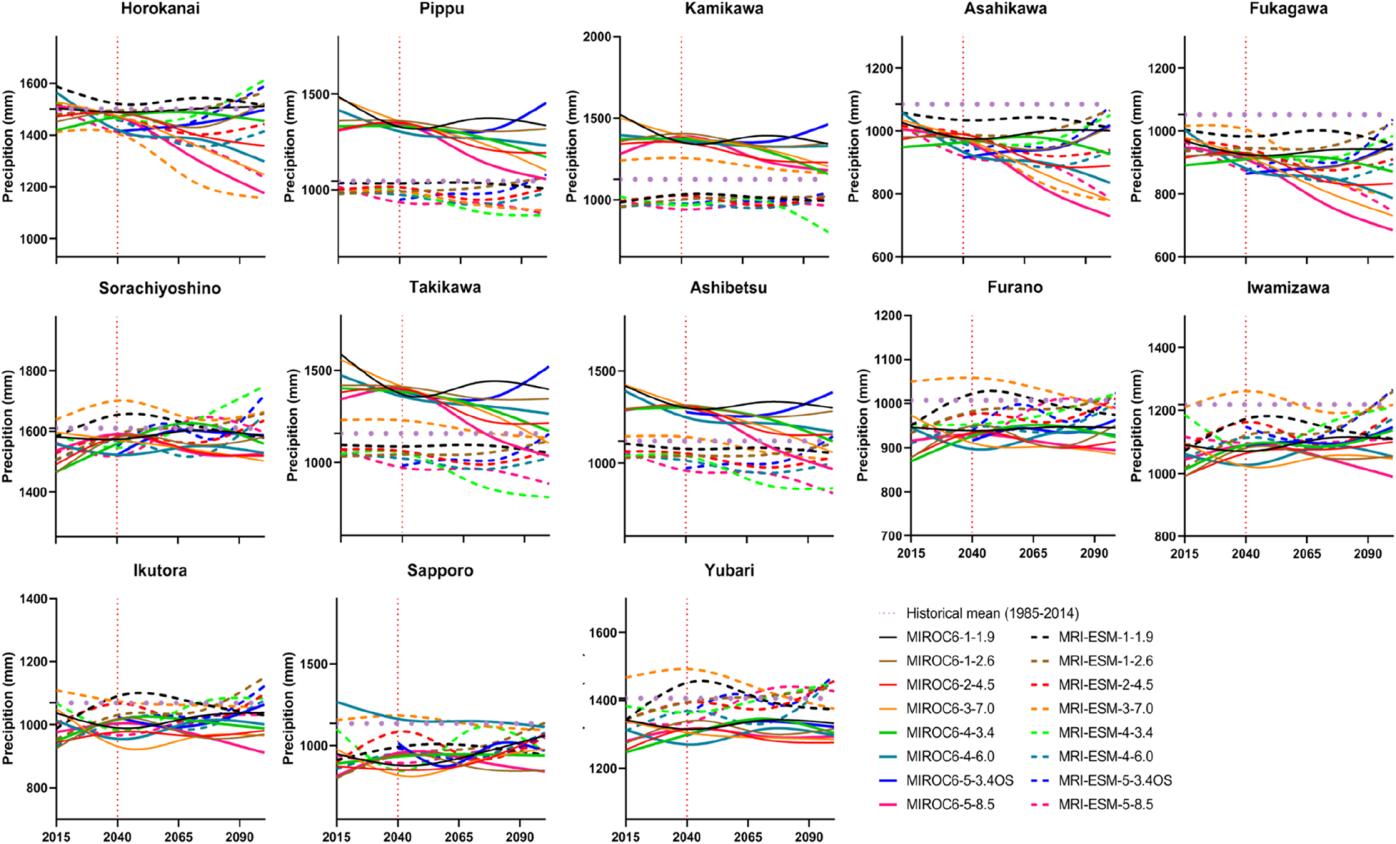
Annual mean precipitation under SSP-RCPs scenarios of two GCMs.

Observed variables in the historical period (1985–2014) were compared to selected GCMs (MIROC6 and MRI-ESM-2.0). Each GCM has eight SSP-RCPs scenarios, SSP1-1.9, SSP1-2.6, SSP2-4.5, SSP3-7.0, SSP4-3.4, SSP4-6.0, SSP5-3.4OS, and SSP5-8.5. Figures 5, 6, and 7 presented projected annual changes (2015–2100) in mean maximum temperature, minimum temperature, as well as precipitation, respectively, at 13 meteorological stations over the IRB. As illustrated in Figs. 5 and 6, there is a noticeable warming trend in the IRB under all scenarios. SSP5-8.5 may force the most severe warming effect in the future, while SSP1-1.9 may have the least. While compared to the observed tasmax of the reference period, the tasmax under SSP1-1.9 was estimated to increase by 1.72–3.47 °C in the 2040s and 1.72–3.53 °C in the 2070s (MRIOC6) and rise by 1.97–3.70 °C in the 2040s and 1.81–3.53 °C in the 2070s (MRI-ESM-2.0). In the projection of SSP5-8.5 scenario, the tasmax may ascend by 2.94–4.58 °C in the 2040s and 5.50–7.19 °C in the 2070s under MRIOC6, and climb by 3.30–5.12 °C in the 2040s and 4.82–6.74 °C in the 2070s under MRI-ESM-2.0, compared to the reference phase. Meanwhile, the tasmin was predicted to involve fewer variations of warming compared to tasmax at all meteorological stations. It was found that tasmin were simulated to increase under SSP5-8.5 of MRIOC6 (2040s: 2.22–4.87 °C; 2070s: 4.08–6.81 °C) and MRI-ESM-2.0 (2040s: 2.60–5.16 °C; 2070s: 3.88–6.48 °C). As for SSP1-1.9, in the weakest scenario, the tasmin went up by 1.31–3.89 °C in the 2040s and 1.30–3.93 °C in the 2070s (MRIOC6), and increased by 1.53–4.05 °C in the 2040s and 1.35–3.90 °C in the 2070s (MRI-ESM-2.0), as relative to the reference stage. Whilst, temperature change in the far-future period (2070s) is anticipated to exhibit greater changes than that in the middle period (2040s). The average temperature was projected to rise by 2.04–4.52 °C in the 2040s and by 2.67–4.94 °C in the 2070s under all scenarios.

Annual variations in precipitation showed more discrepancies not only at different meteorological stations but also under different SSP-RCPs scenarios from two GCMs (Fig. 7). For example, at the Pippu, Kamikawa, Takikawa, and Ashibetsu stations, SSP-RCPs scenarios from MIROC6 generated higher annual precipitation than those in MRI-ESM-2.0. But at other stations such as Horokanai, Scenario SSP1-1.9 from MRI-ESM-2.0 could predict more precipitation than that in MRIOC6. As shown in Fig. 7, annual precipitation was expected to drop at most stations. With the increase in CO_2_ emissions, the precipitation presented a tendency opposite to that of temperature. Precipitation will reduce with the temperature rising in the IRB. Particularly, precipitation in the far-future phase was predicted to decrease more than that in the middle period. Under all scenarios of MIROC6, the mean annual precipitation is likely to decrease by 2–17% during the 2040s, except that the Pippu (25%), Kamikawa (20%), Takikawa (17%), and Ashibetsu (13%) stations, respectively, while during the 2070s, it may reduce by 7–20%, except that the Pippu, Kamikawa, Takikawa, and Ashibetsu stations may have their precipitation increased by 19%, 15%, 10%, and 7%, respectively. Under all scenarios of MRI-ESM-2.0, the reduction of rainfall is expected to reach 0.1–15% (2040s) and 0.02–17% (2070s), respectively.

### Projection of climatic variables

**Figure 8:**
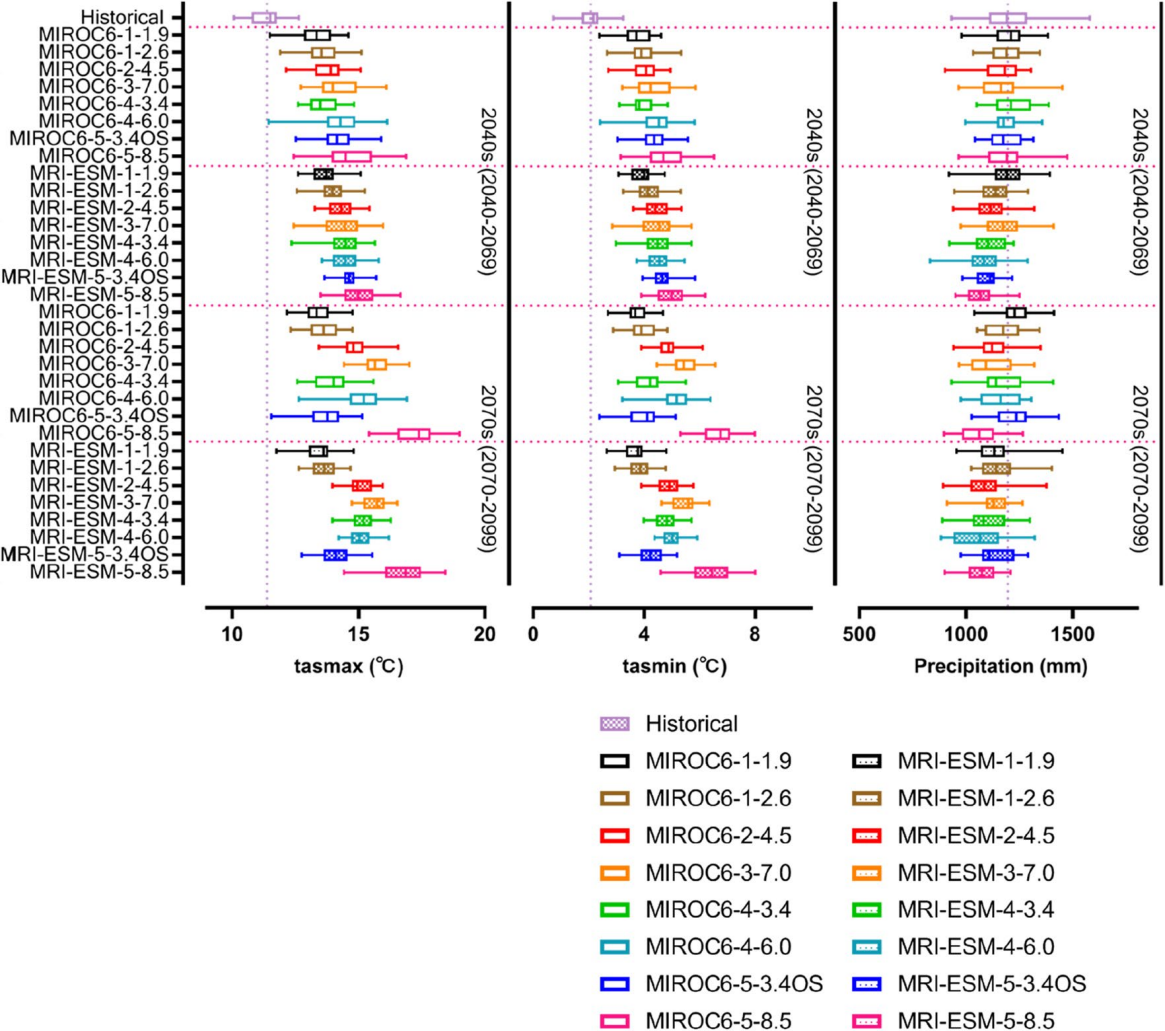
Variations in maximum temperature (tasmax), minimum temperature (tasmin), and precipitation in periods of the 2040s and 2070s under SSP-RCPs scenarios of MIROC6 and MRI-ESM-2.0.

**Figure 9:**
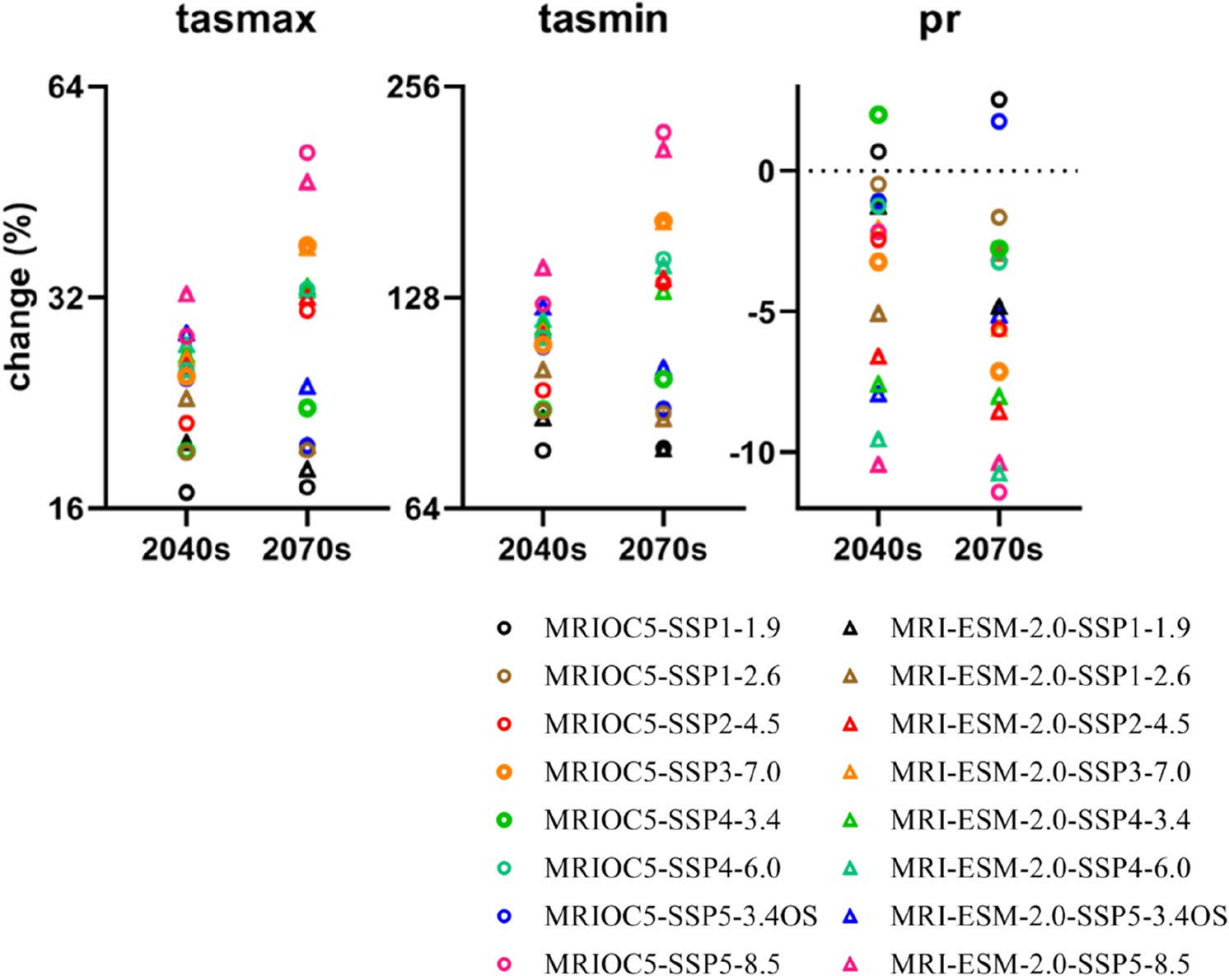
Percent change of average maximum temperature (tasmax), minimum temperature (tasmin), and precipitation in the 2040s and 2070s, with respect to the baseline (1985–2014).

Figures 8 and 9 showed the variations in average climatic variables (tasmax, tasmin, and precipitation) of all meteorological stations under MIROC6 and MRI-ESM-2.0 scenarios in two phases of 2040s and 2070s, in respect to the reference stage of 1985–2014. The SSP-RCP scenarios had a clear impact on temperature and rainfall. Scenario SSP5-8.5 always generated the greatest warming trend with the tasmax and tasmin increasing under MRIOC6 (4.53 °C and 3.59 °C) and MIR-ESM-2.0 (4.50 °C and 3.63 °C) respectively, comparing to baseline (Fig. 8). Following the SSP5-8.5, SSP3-7.0, SSP4-6.0, SSP4-3.4, SSP2-4.5, SSP5-3.4OS, and SSP1-2.6 scenarios were also likely to increase temperature. The scenario SSP1-1.9 projected the least increase in tasmax (MIROC6: 17.1%; MRI-ESM-2.0: 19.1%) and tasmin (MIROC6: 77.8%; MRI-ESM-2.0: 82.0%) (Fig. 9). The emission scenario had a greater impact on temperature and precipitation projection than the socioeconomic scenario. The increasing range is from the high-emission (SSP5-8.5) to the low-emission case (SSP1-1.9). Those results correspond to the original setting of each scenario of CMIP6.

In direct contrast, precipitation exhibited opposite variations under SSP-RCP scenarios compared to tasmax and tasmin. Under MRIOC6, SSP4-3.4 (24.1 mm) produced the greatest increase of rainfall and SSP3-7.0 (− 38.7 mm) made the least in 2040s, SSP1-1.9 (30.3 mm) generated the highest amount of rainfall and SSP5-8.5 (− 136.5 mm) made the greatest decrease in 2070s. Under MIR-ESM-2.0, SSP1-1.9 (− 15.0 mm) generated the greatest amount of rainfall and SSP5-8.5 (− 124.8 mm) made the least in 2040s, SSP1-2.6 (− 35.0 mm) produced the greatest amount of rainfall and SSP4-6.0 (− 128.5 mm) made the least in 2070s (Fig. 8). In the comparison of each SSP-RCPs scenario, SSP1-1.9 always produced the highest amount of rainfall, with the percent changes of − 0.3% (mean value of two GCMs) in the 2040s and − 1.1% in the 2070s, and SSP5-8.5 made the least, with the percent changes of − 6.3% (2040s) and − 10.9% (2070s) (Fig. 9). In addition, MIROC6 could support a wider range of variations in maximum and minimum temperatures than MRI-ESM-2.0. The percent changes under MIROC6 were 16.9–51.5% for tasmax and 77.5–220.3% for tasmin, respectively. In terms of MRI-ESM-2.0, variations were from 18.2 to 46.8% for tasmax and from 77.8 to 208.0% for tasmin, respectively. In general, the average temperature under all scenarios of MIROC6 and MRI-ESM-2.0 was anticipated to rise by the range of 1.89–3.10 °C during the 2040s and by 1.81–5.02 °C during the 2070s. The precipitation decreased by − 11.4–2.5% (MIROC6) and − 10.8%–− 1.3% (MRI-ESM-2.0).

**Figure 10:**
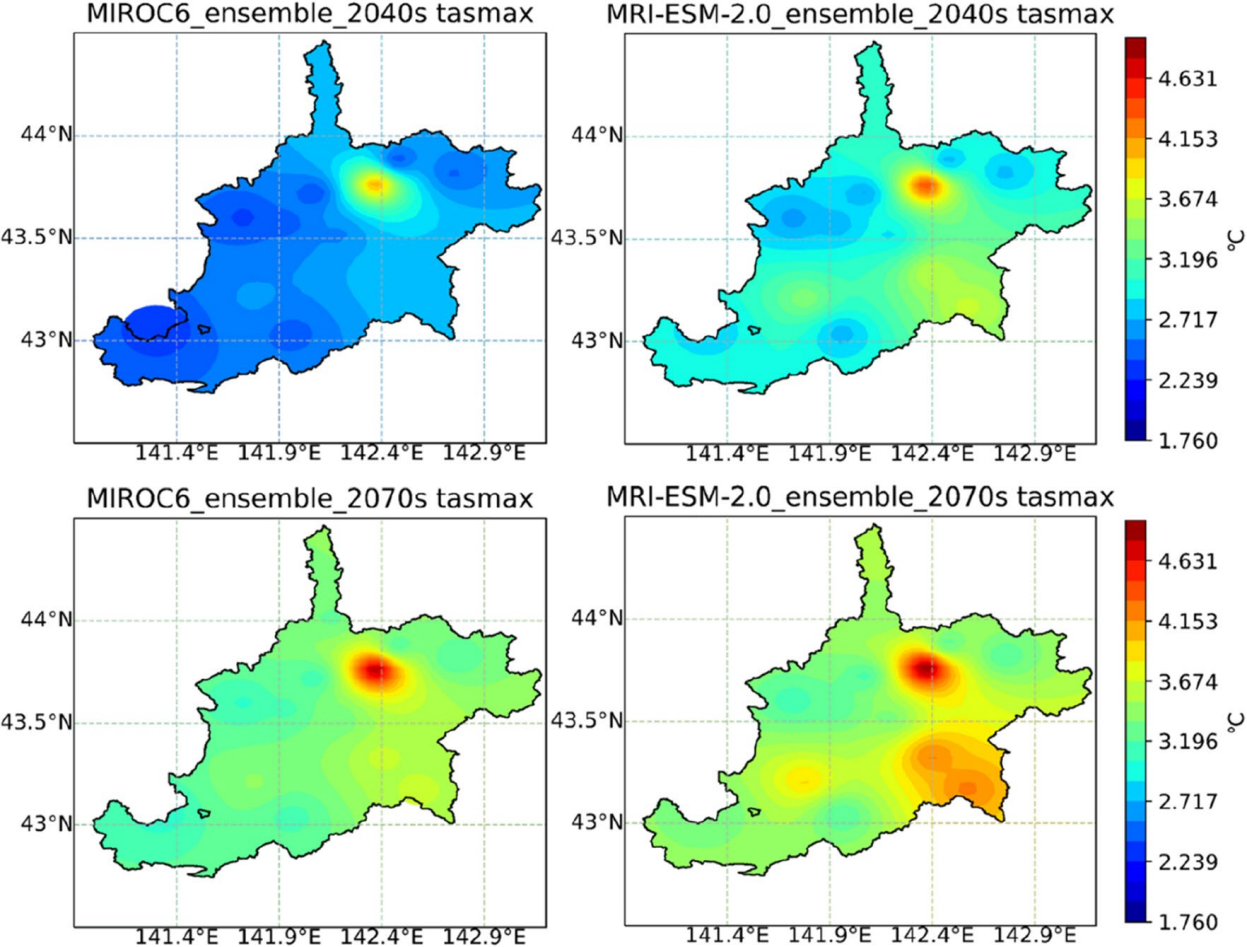
Changes in the maximum temperature (tasmax) of the Ishikari River basin in periods of the 2040s and 2070s under MIROC6 and MRI-ESM-2.0.

**Figure 11:**
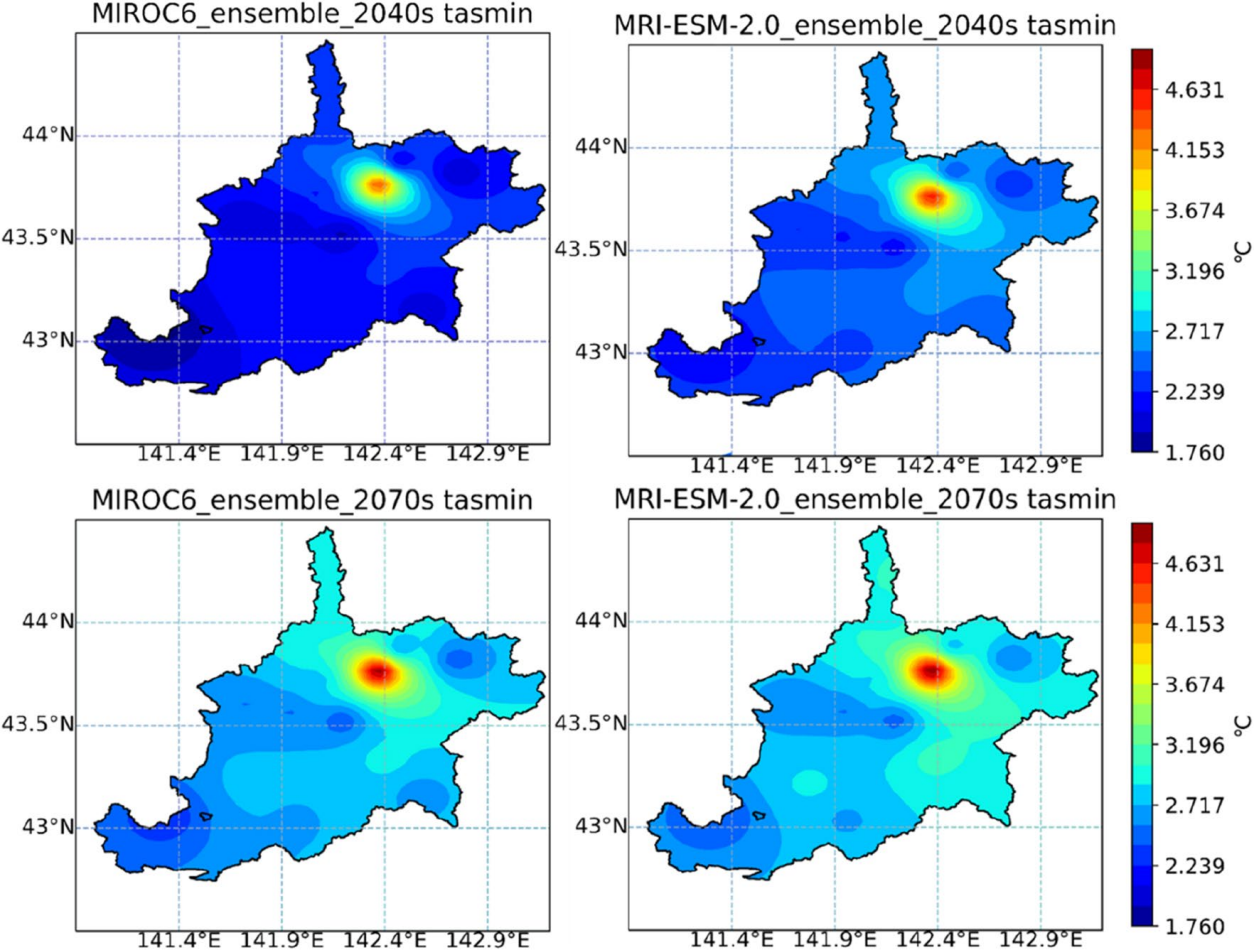
Changes in the minimum temperature (tasmin) of the Ishikari River basin in periods of the 2040s and 2070s under MIROC6 and MRI-ESM-2.0.

**Figure 12:**
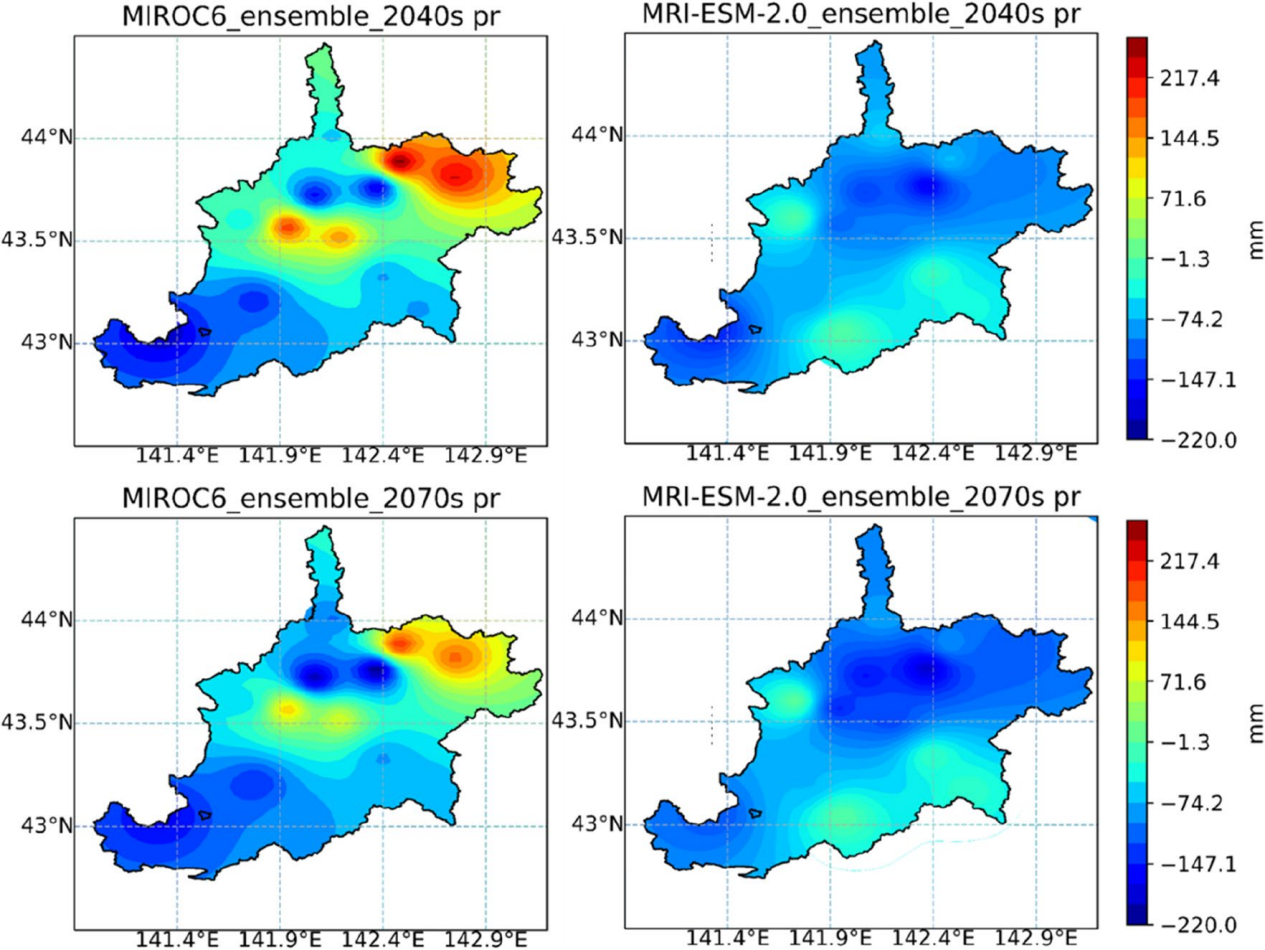
Changes in the annual precipitation (pr) of the Ishikari River basin in periods of the 2040s and 2070s under MIROC6 and MRI-ESM-2.0. Note: Figs. 10, 11 and 12 were generated by Jupyter Notebook (https://jupyter.org/) based on Python 3.8.3. Shapefile of Ishikari River basin is download from the Japanese Geographical Survey Institute (JGSI, http://nlftp.mlit.go.jp).

The distribution of average changes in maximum temperature, minimum temperature, as well as precipitation of the IRB, during the 2040s and 2070s under the MIROC6 and MRI-ESM-2.0, are displayed in Figs. 10, 11, and 12, respectively. MIROC6 and MRI-ESM-2.0 both show the higher air temperature and less precipitation (Figs. 10, 11, and 12). Spatially, when it came to the distribution of tasmax and tasmin, both products showed a similar trend, with a bigger rise in the northern part and a smaller rise in the southern part of the IRB. According to meteorological data, the northern part was colder than the southern part of the study area, but CMIP6 GCMs anticipated that the northern part may exhibit a stronger warming trend in the future. The precipitation across the IRB showed a decreasing trend under MRI-ESM-2.0. However, a large increase in precipitation produced by MRIOC6 was found in the mid-northern part of the IRB, which was displayed as an absolute difference from MRI-ESM-2.0. Temporally, higher temperature and less precipitation were projected during the late twenty-first century (2070s) than the mid-century (2040s) in the IRB. The distribution of climatic variable changes was strongly affected by emissions. For all SSP-RCP scenarios assessment, the distribution of climatic variables change was strongly affected by emissions (Supplementary Figs. 1–3). Higher emissions are associated with higher temperatures and less precipitation under all of the climatic scenarios in this study.

## Discussion

In this study, we compared climatic variables (air maximum/minimum temperature and precipitation) from 17 available CMIP6 GCMs at 13 meteorological stations in the IRB. The performances of 17 GCMs were evaluated by Taylor diagrams with two evaluation metrics (correlation coefficient and root-mean-square difference). Most CMIP6 GCMs were able to access temperature measurements with high correlation coefficients. But there is not a single GCM could well reproduce the precipitation characteristics across the IRB. Our results showed only two preferred GCMs, i.e., MIROC6 and MRI-ESM-2.0 showed the best adaptability in temperature and precipitation across the target region.

Accordingly, in order to generate daily maximum/minimum temperature and daily precipitation in two future phases, the 2040s (2040–2069) and 2070s (2070–2099), a statistical downscaling model was established based on the 20CRv3 reanalysis datasets and CMIP6 GCMs-derived predictors, with respect to observed climate during 1985–2014. Constructed SDSM had satisfactory modeling performance in both temperature and precipitation throughout calibration (1985–1999) and validation (2000–2014) stages (R^2^, NSE > 0.957). SDSM presented a better ability in simulating temperature than precipitation. CMIP6 GCMs showed a significant correlation with temperature measurements, but were unable to represent rainfall features in the IRB. Detecting precipitation features derived from climate model simulations is more strenuous relative to temperature^54^. No CMIP3 model could recreate the magnitude of the seasonal precipitation cycle in the western USA^55^. All eleven GCMs from CMIP3 have trouble simulating precipitation in peninsular India^56^. Benedict et al. also found that CMIP5 GCMs could not improve the precipitation budget of the Mississippi basins^14^. That is because GCMs are incapable of modeling precipitation with high accuracy, e.g., on the local station scale, and the GCM outputs of precipitation are affected by topographical factors and regional climatic forcing^23^. On the other hand, the downscaling process of precipitation further propagates this error^57^. It should be more cautious when downscaling precipitation with SDSM^58^. To sum up, FGOALS-g3, CanESM5, MIROC6, and MRI-ESM2-0 have the best predictive ability of maximum temperature, MRI-ESM2-0 has the best predictive ability of minimum temperature, and MIROC6 has the best predictive ability of precipitation. Given the availability of each GCM with adequate predictors for SDSM simulating, two GCMs, i.e., MIROC6 and MRI-ESM2-0, from CMIP6 were chosen to generate future climatic variables under various SSP-RCPs scenarios.

Future climatic variables were projected in all SSP-RCP scenarios (SSP1-1.9, SSP1-2.6, SSP2-4.5, SSP3-7.0, SSP4-3.4, SSP4-6.0, SSP5-3.4OS, and SSP5-8.5). Average tasmax and tasmin under eight SSP-RCP scenarios were predicted to rise about 1.92–5.85 °C and 1.61–4.58 °C (MIROC6), 2.07–5.32 °C and 1.62–4.32 °C (MRI-ESM-2.0), respectively (Fig. 8). The average temperature of two GCMs was anticipated to rise by 1.89–3.10 °C (2040s) and 1.91–5.02 °C (2070s) under all scenarios. The latest generation simulations, CMIP6, continue to paint a picture of more frequent and intense high temperatures on a wide scale^59^. It's hardly surprising that projected climate enhanced warming during 2040–2099 under all SSP-RCP scenarios. On a global level, the annual mean temperature under CMIP6 shows a more sensitive warming trend at higher latitudes^60^, which indicates that the warming effect in the north region of IRB is greater than that in the southern (Figs. 10 and 11).

Future annual rainfall, generated from MIROC6 and MRI-ESM-2.0, broadly decreased in the projected periods (2040s: − 1.0% and − 6.3%; 2070s: − 3.4% and − 7.0%, respectively) (Fig. 9). When the air temperature rises, the increasing evaporation may be able to remove more water, resulting in more frequent and intense storms or less precipitation and increased risk of drought^61^. In the example of the Ishikari River basin, it may explain that the climate with a greater warming effect from downscaled GCMs derived may cause the less total amount of precipitation over land. Similar findings have appeared in the Duan et al. which revealed the decreased precipitation amount under CMIP3 GCMs in the upper Ishikari River basin^62^, this is consistent with our findings. Meanwhile, the distribution of rainfall under CMIP6 GCMs shows discrepancies in variable rate between the north and south regions in Ishikari River basin. A number of studies indicate that warming will increase precipitation in equatorial and high-latitude regions^63,64^. Generally, our finding suggested that more amounts of precipitation would appear in the northern than southern of the IRB (Fig. 12).

However, uncertainties in climate models are inevitably subjected to prove the limiting factors, contributing to model structures^65,66^, downscaling approaches^67,68^, and local forcing, even for very broad regional averages^69^. When further employing the GCMs-derived datasets, how to reduce this uncertainty is not unavoidably overlooked. Different climate models are constructed based on sophisticated amalgamations of various patterns and assumptions, which would lead to large uncertainties in this work of climate projections^70^. But several studies have exemplified that the multi-model ensemble could be an efficient method to lower model-specific uncertainty^71^. Here 17 available CMIP6 GCMs and eight scenarios were employed to construct the future climate (temperature and precipitation) in the IRB. CMIP6 GCMs were selected by evaluating model applicability with several metrics. Preferred multi-GCMs and SSP-RCP scenarios could provide a wider variation range of climatic variables to eliminate further uncertainties when projecting future climate change scenarios^72^. The findings of our work could support the future projections of essential climatic variables under all the SSP-RCP scenarios. The uncertainty envelope was also largely shaped by the downscaling techniques. There should be caution if only one approach is used to reduce uncertainty in downscaling progress. Particularly, using the regression-based methods (such as the SDSM application in this study) would raise the concern of the anomalous downscaled future temperatures increasing^73^.

Besides forcings of model and downscaling, this, specific geographic location of the IRB, in particular, makes precise precipitation distribution and amount predictions difficult^74^. Due to the topography of the coast and the complexity of orographic effects (Mashike Mountains and Taisetsu Mountains). During the Asian winter monsoon, sea-effect snowfall generated over the Sea of Japan affects the Ishikari River basin^75,76^. In addition, several efforts on climate modeling have pointed out that global warming causes more intense precipitation but reduced precipitation frequency^63^. Since precipitation is such complex combinations of different physical processes, the volume of precipitation is determined not only by the severity but also by the frequency^77^. In this study, we just estimated the amount of precipitation, lacking other characteristics of precipitation (e.g., intensity and frequency), further study is needed to properly elucidate the influence of global warming on the internal variability of the geophysical system, and then to fully understand how climate change drives the mechanisms that result in extreme events^71^. Since the climate with warming and less rainfall would pose a significant challenge to ecosystems of IRB, some studies show rising temperature can increase crop yield^78^. Groundwater flow is also affected by precipitation when less streamflow caused by rainfall decreasing^79^. Those fundamental datasets would be dedicated to further climate change related work, such as modeling hydrological processes, simulating crops growth and yield, evaluating ecosystem services, and so on. Those potential situations should be considered in further studies when developing models.

## Conclusion

In this study, reconstructed SDSM was successfully developed using the datasets from the 20CRv3 and two preferred GCMs (MIROC6 and MRI-ESM-2.0). Then, future climatic variables were downscaled under all SSP-RCP scenarios (SSP1-1.9, SSP1-2.6, SSP2-4.5, SSP3-7.0, SSP4-3.4, SSP4-6.0, SSP5-3.4OS, and SSP5-8.5) from MIROC6 and MRI-ESM-2.0, respectively. Downscaled projections based on CMIP6 models are likely to generate the warmer and dryer climate over Ishikari River basin. Increased temperature and less precipitation changes in the far-future period (2070s) may be larger than those in the middle period (2040s). Hence, our results forecast plausible future climate change. The datasets of climatic variables established in this study can be utilized in regional or local hydrologic and environmental modeling, as well as in analyzing the sustainability of ecosystems in further research.

## Supplementary Information

Supplementary Figures.

## Data Availability

All meteorological datasets used in this study are provided by the Japan Meteorological Agency (JMA, http://www.jma.go.jp). The 20CRv3 datasets are available at the NOAA Physical Sciences Laboratory (PSL, https://www.psl.noaa.gov/). The daily atmospheric predictors are accessed from the Coupled Model Intercomparison Project Phase 6 (CMIP6, https://esgf-node.llnl.gov/search/cmip6/).
