# Supplementary material for: Climate change multi-model projections in CMIP6 scenarios in Central Hokkaido, Japan
Source: Sci Rep. 2023 Jan 5;13:230. doi: 10.1038/s41598-022-27357-7 (PMC9816114; doi:10.1038/s41598-022-27357-7)
Supplement: Supplementary file 1 — Supplementary Figures. [file 41598_2022_27357_MOESM1_ESM.doc]

**
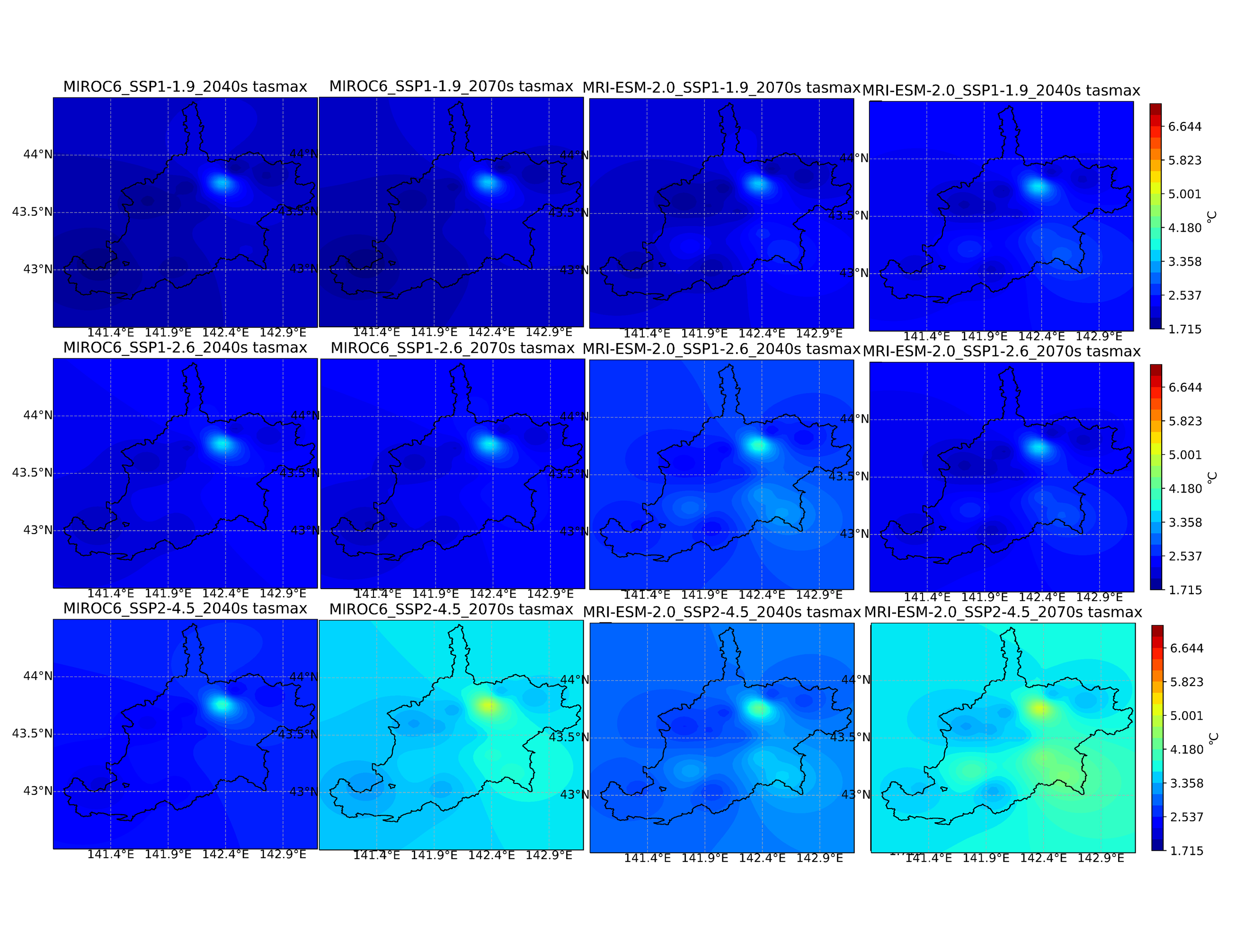
**

**
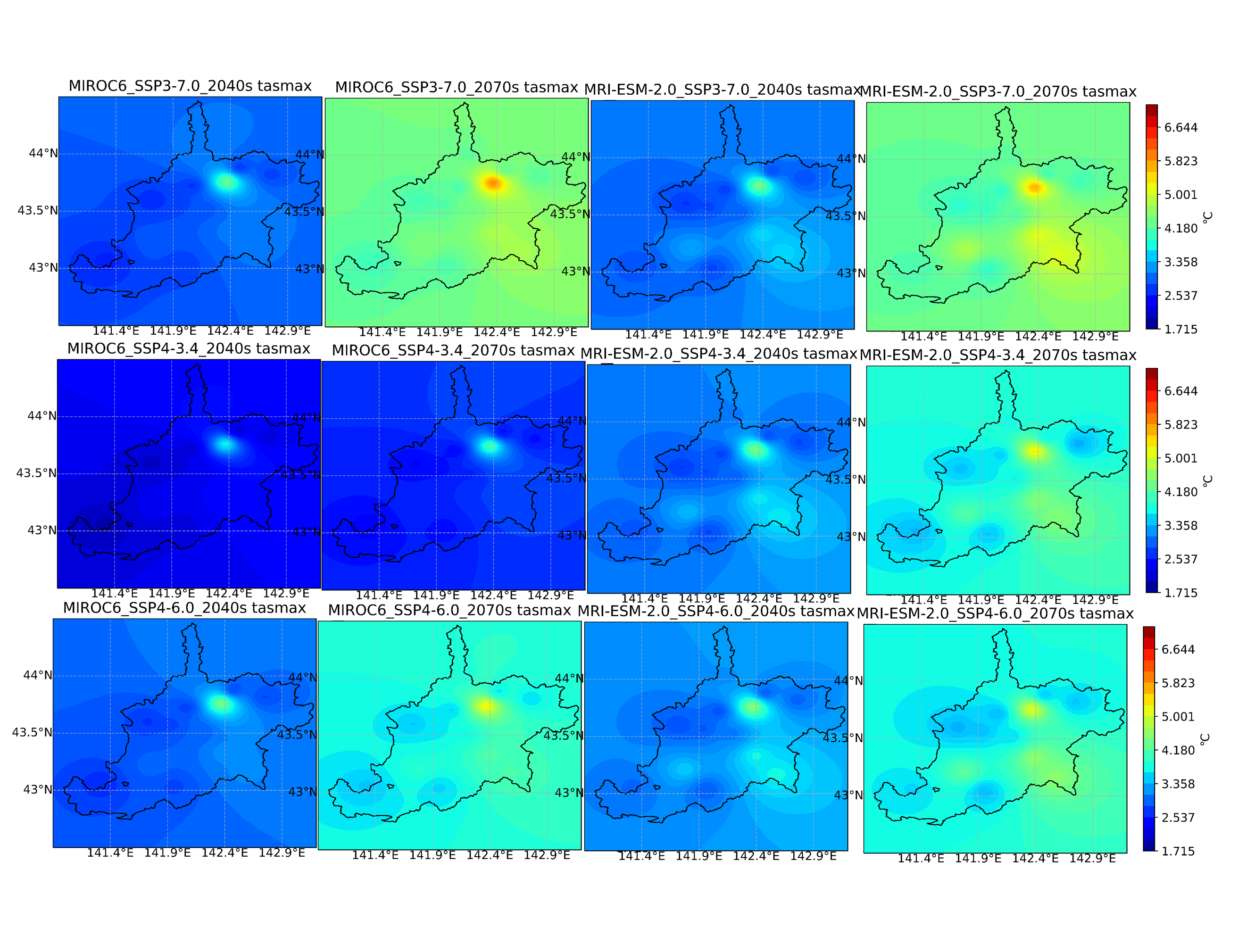
**

**
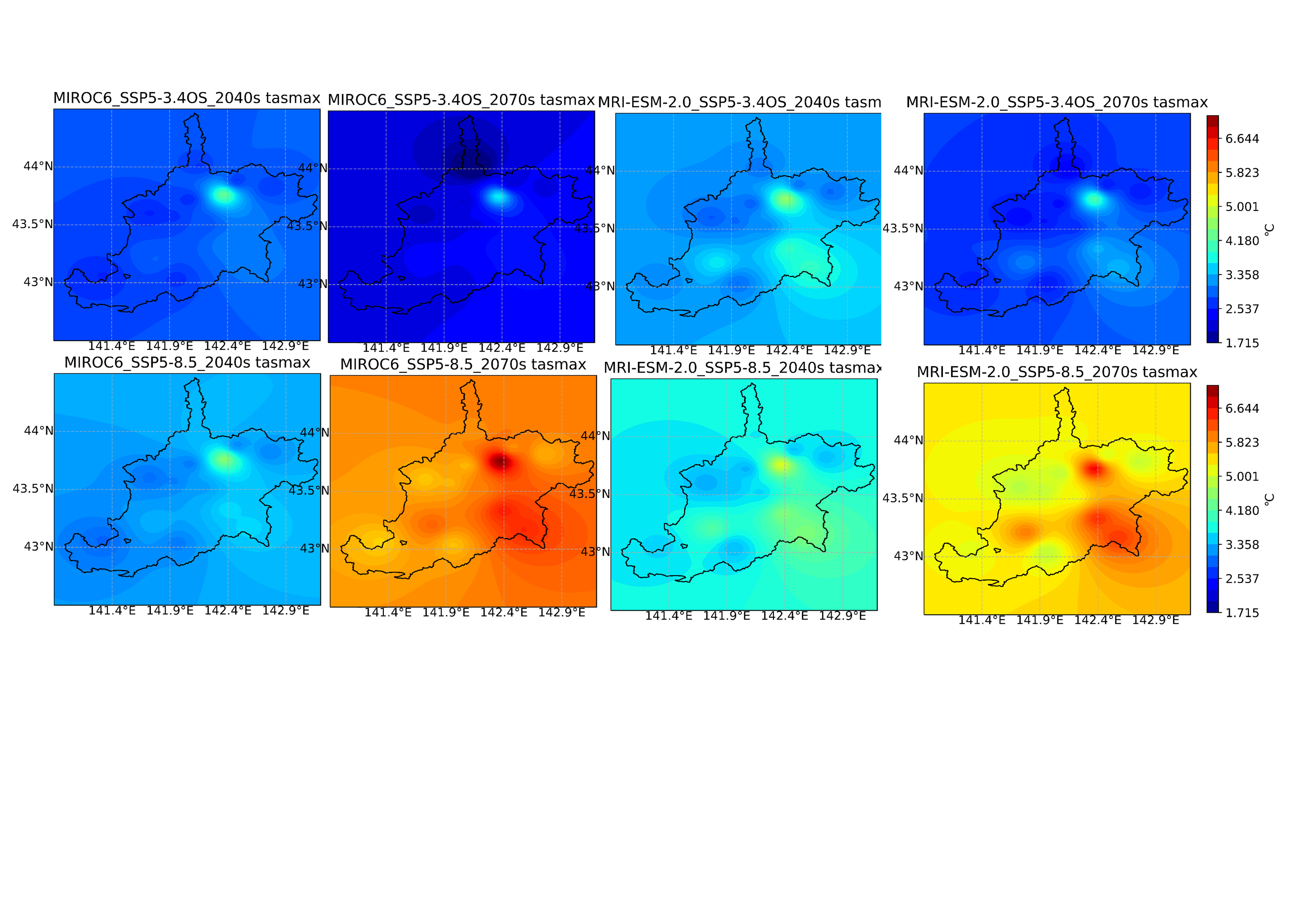
**

**Supplementary Figure 1 Change of maximum temperature (tasmax) map for Ishikari River Basin under SSP-RCP scenarios in periods of 2040s and 2070s relative to the reference period**

**
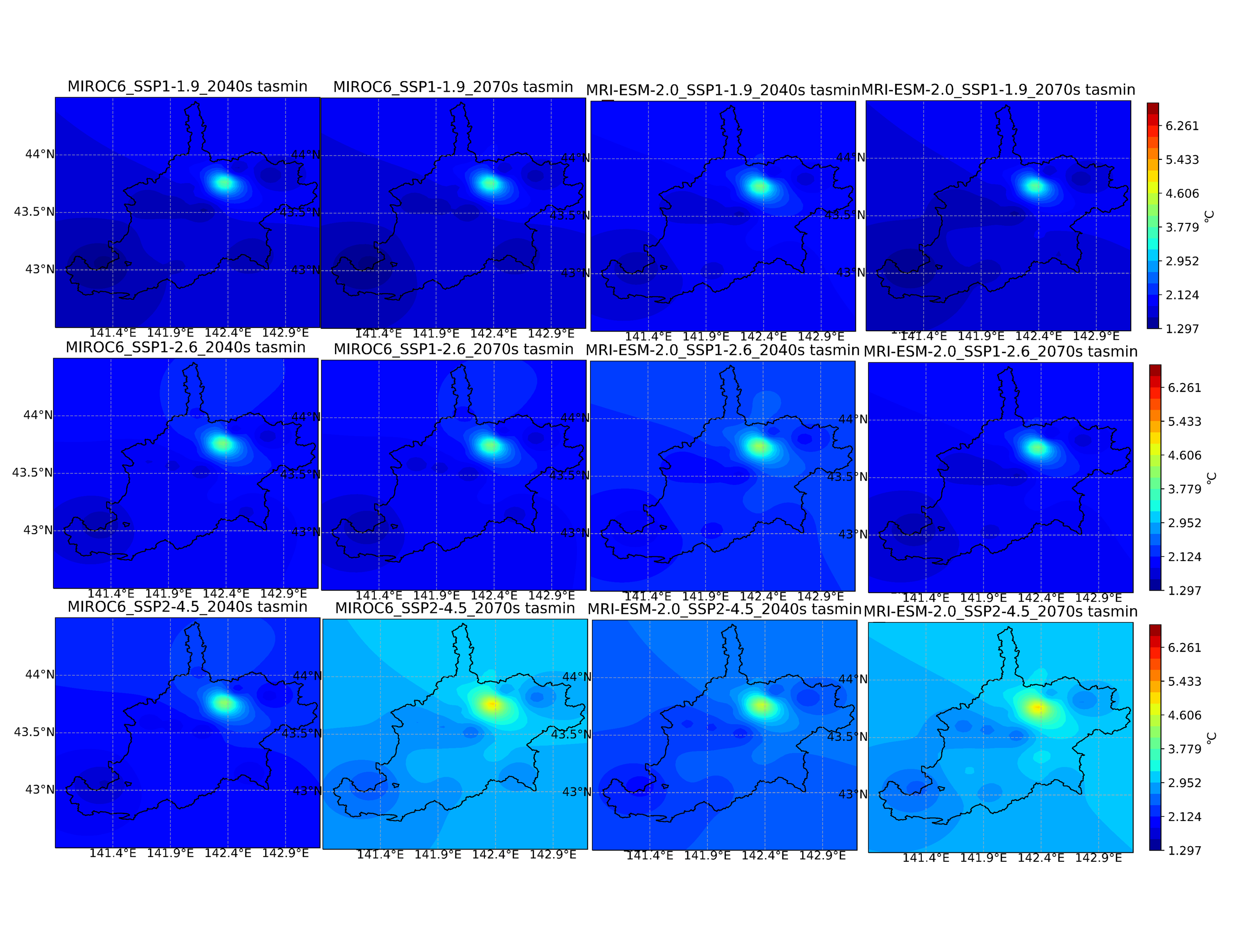
**

**
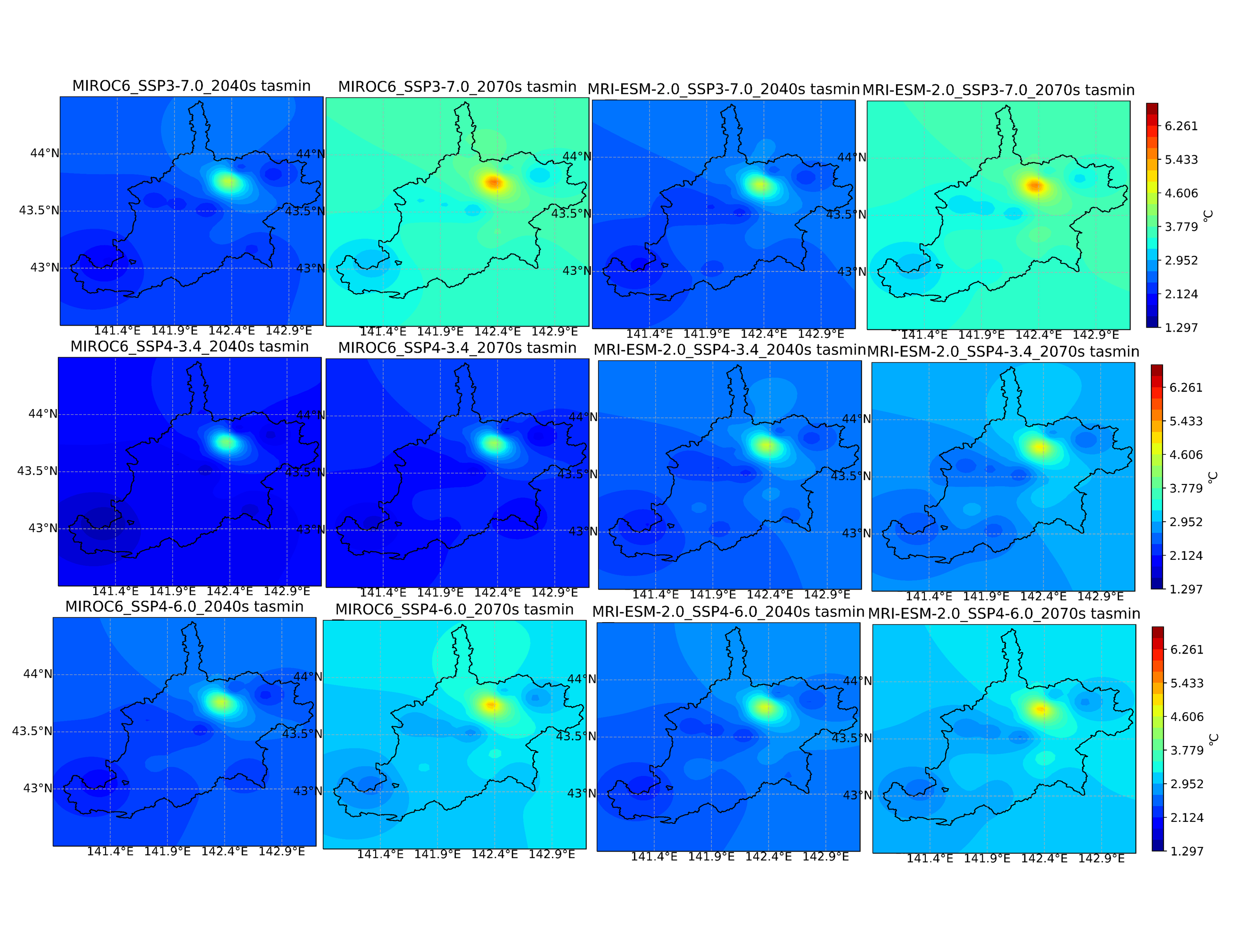
**

**
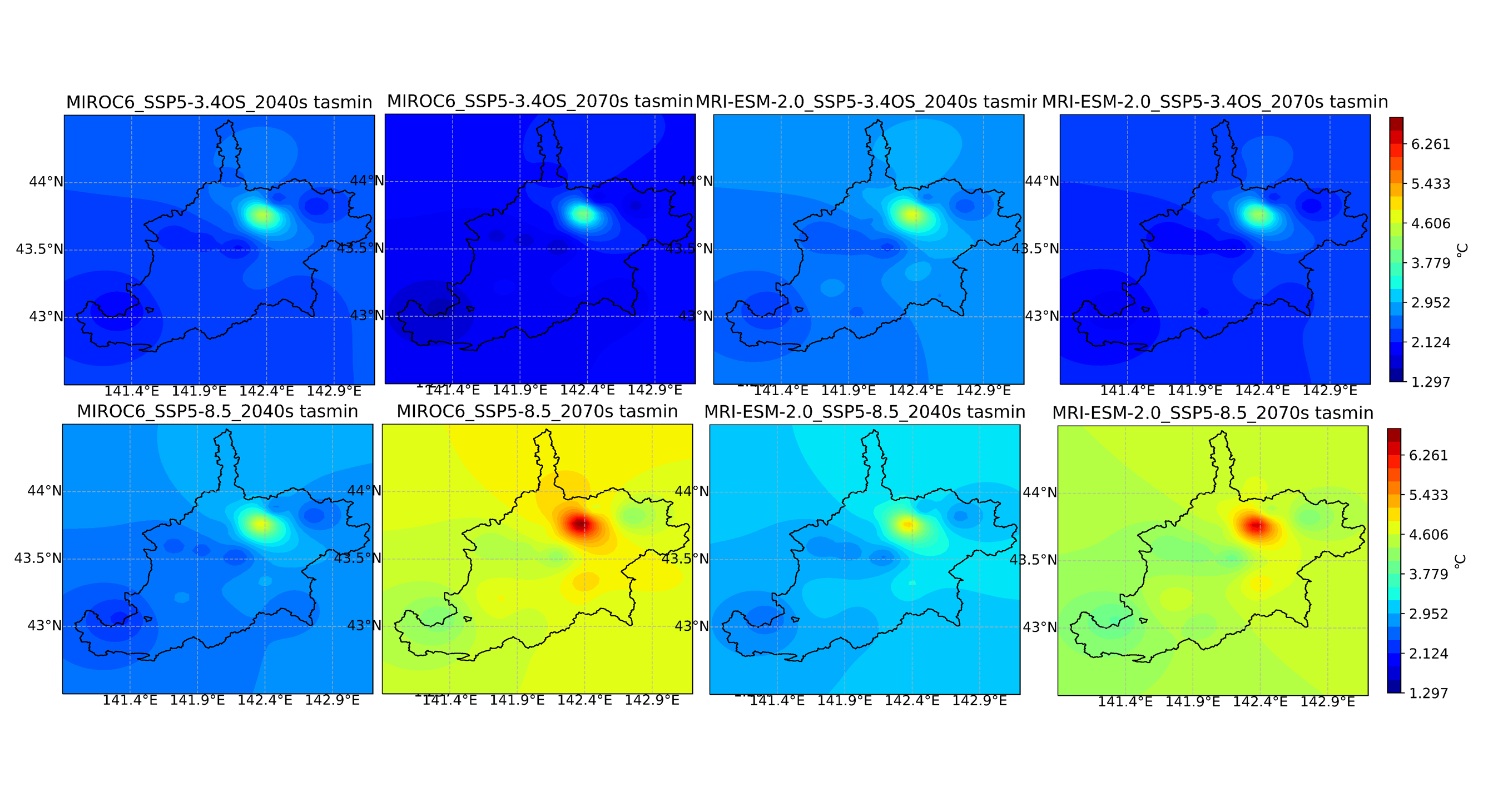
**

**Supplementary Figure 2 Change of minimum temperature (tasmin) map for Ishikari River Basin under SSP-RCP scenarios in periods of 2040s and 2070s relative to the reference period**

**
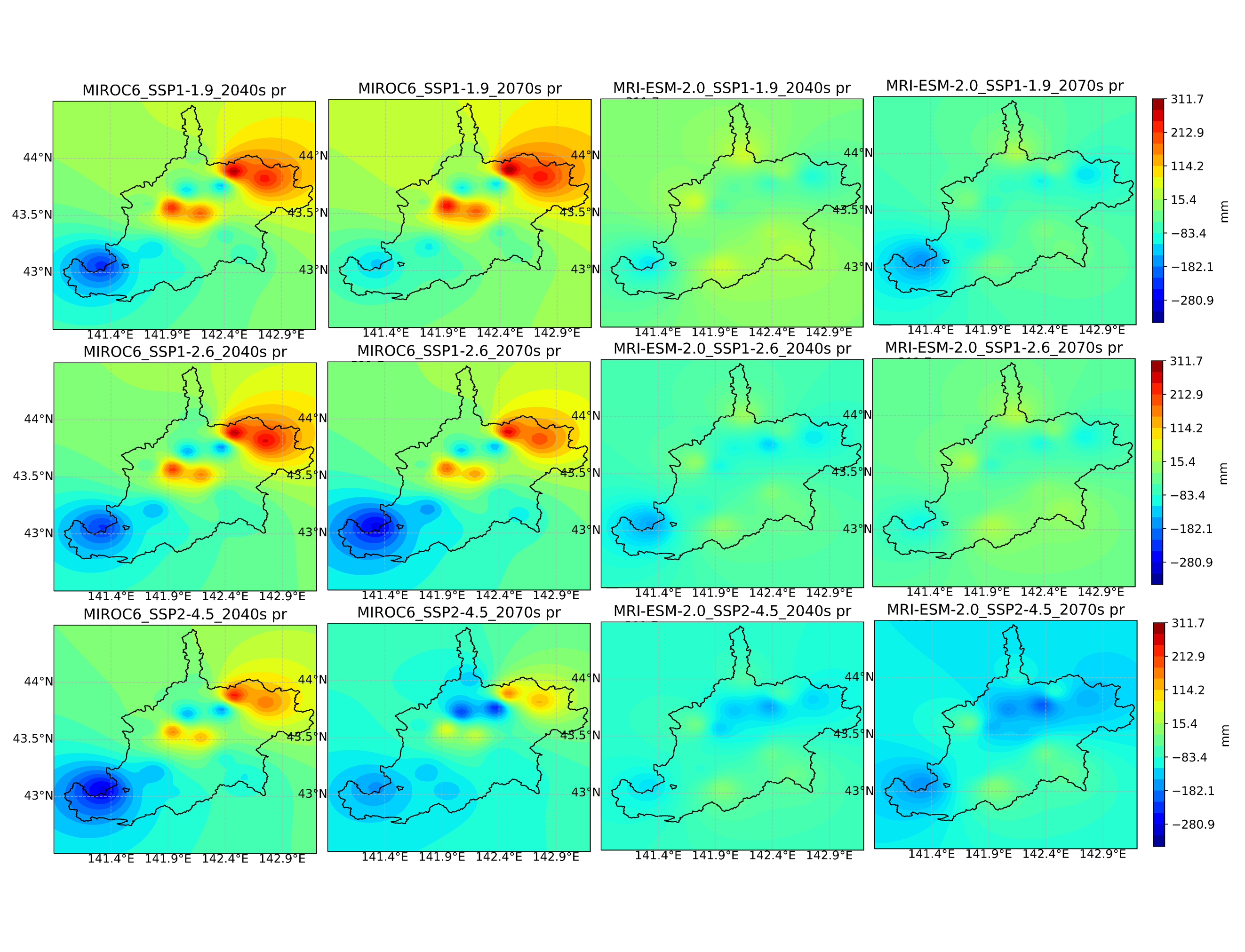
**

**
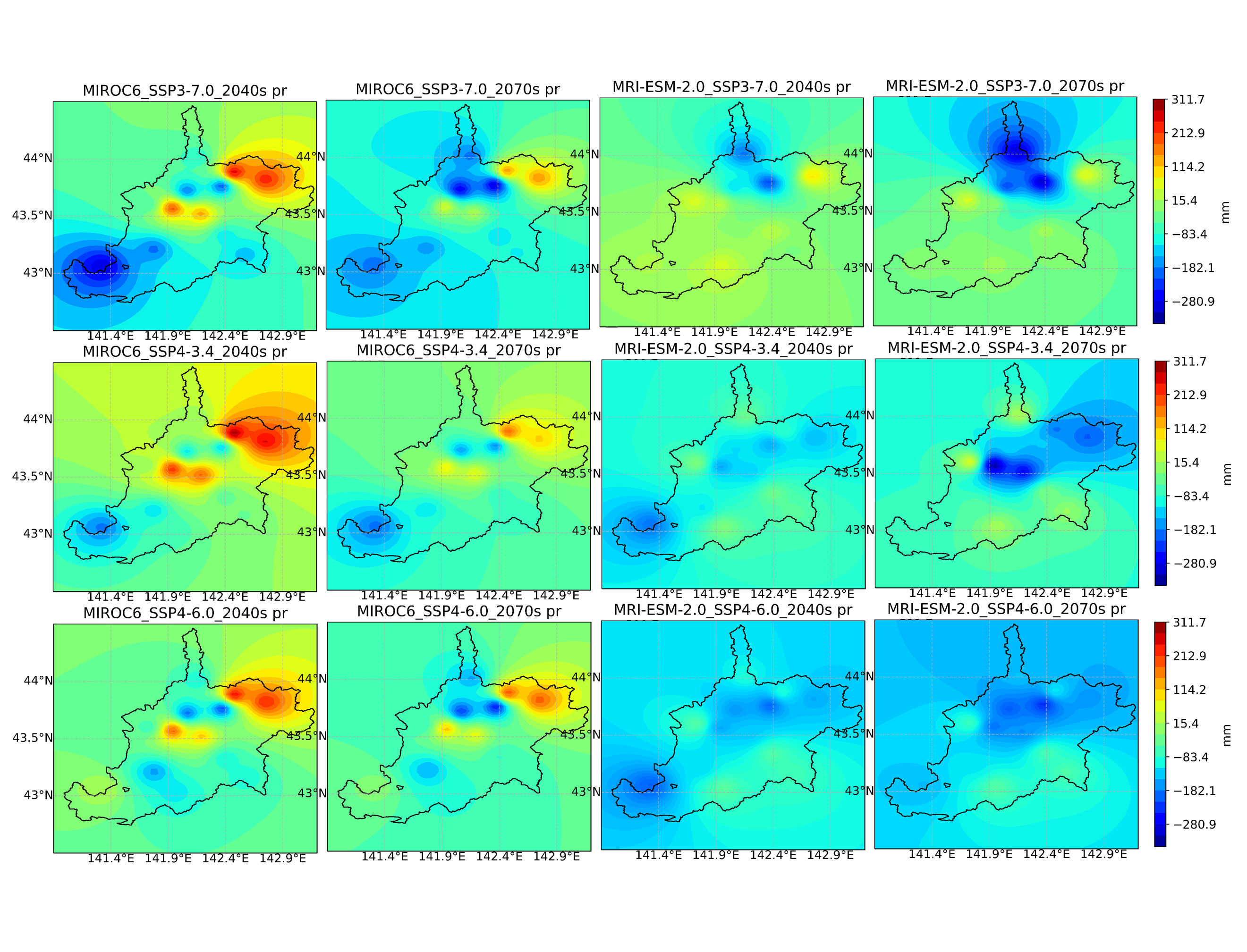
**

**
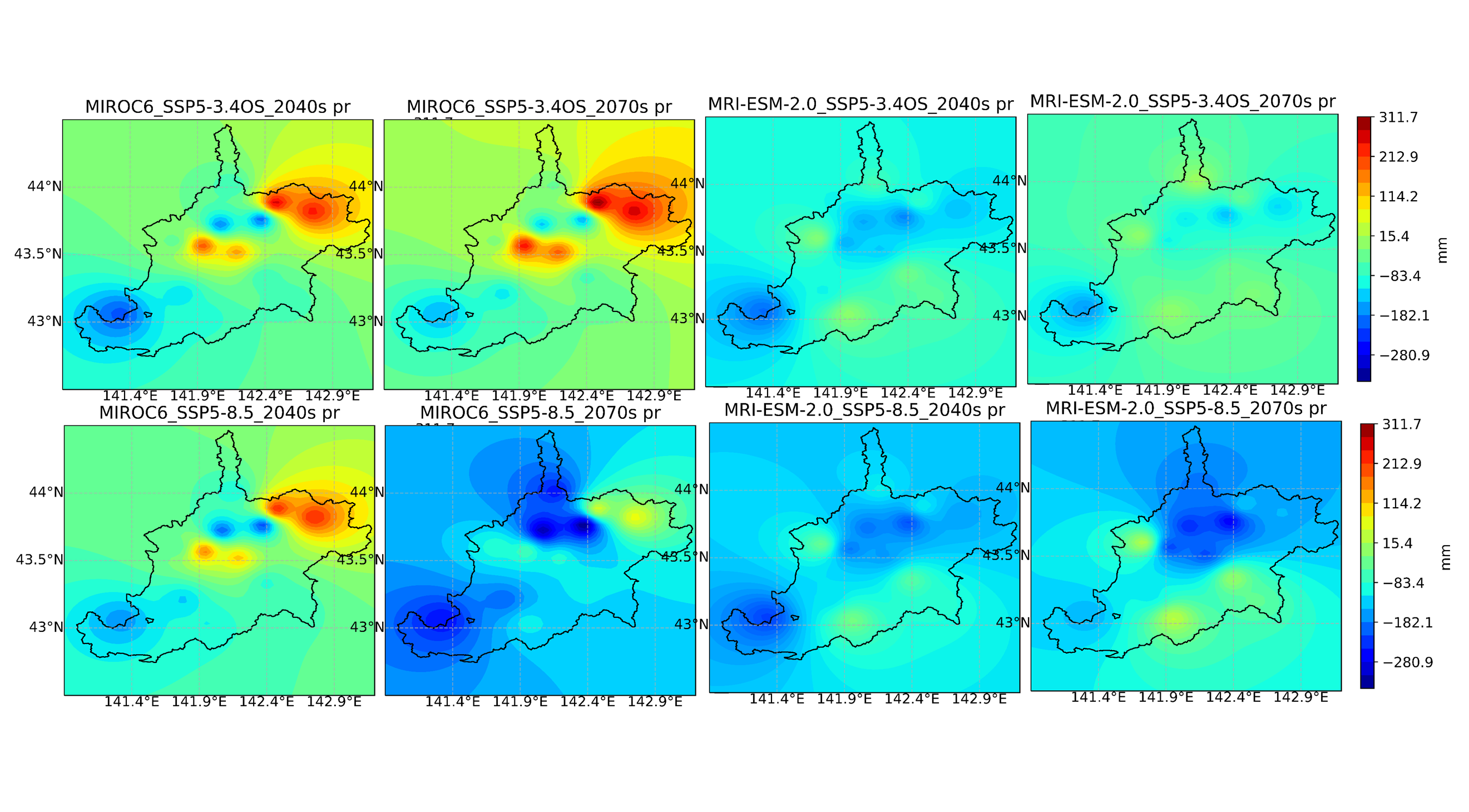
**

**Supplementary Figure 3 Change of precipitation (pr) map for Ishikari River Basin under SSP-RCP scenarios in periods of 2040s and 2070s relative to the reference period**

Note: Supplementary Figures 1, 2 and 3 were generated by Jupyter Notebook (https://jupyter.org/) based on Python 3.8.3. Shapefile of Ishikari River basin is download from the Japanese Geographical Survey Institute (JGSI, http://nlftp.mlit.go.jp).
